# Pharmacogenomic Analyses Implicate B Cell Developmental Status and *MKL1* as Determinants of Sensitivity toward Anti-CD20 Monoclonal Antibody Therapy

**DOI:** 10.3390/cells12121574

**Published:** 2023-06-07

**Authors:** George W. Small, Farida S. Akhtari, Adrian J. Green, Tammy M. Havener, Michael Sikes, Julia Quintanhila, Ricardo D. Gonzalez, David M. Reif, Alison A. Motsinger-Reif, Howard L. McLeod, Tim Wiltshire

**Affiliations:** 1 Pharmacotherapy and Experimental Therapeutics, University of North Carolina at Chapel Hill, Chapel Hill, NC 27599, USA; george_small@med.unc.edu (G.W.S.); rgonzalez1@unc.edu (R.D.G.); 2 Biostatistics and Computational Biology Branch, Division of Intramural Research, National Institute of Environmental Health Sciences, Research Triangle Park, NC 27709, USA; farida.akhtari@nih.gov (F.S.A.);; 3 Department of Biological Sciences, Bioinformatics Research Center, North Carolina State University, Raleigh, NC 27695, USA; 4 Structural Genomics Consortium and Division of Chemical Biology and Medicinal Chemistry, Eshelman School of Pharmacy, University of North Carolina at Chapel Hill, Chapel Hill, NC 27599, USA; thavener@unc.edu; 5 Department of Biological Sciences, North Carolina State University, Raleigh, NC 27695, USA; mlsikes@ncsu.edu; 6 Clinical Development, Foundation Medicine, Boston, MA 02141, USA; jcfquintanilha@gmail.com; 7 Predictive Toxicology Branch, Division of Translational Toxicology, National Institute of Environmental Health Sciences, Research Triangle Park, NC 27709, USA; david.reif@nih.gov; 8 Center for Precision Medicine and Functional Genomics, Utah Tech University, 225 South University Ave, St. George, UT 84770, USA; hmcleod1965@gmail.com; 9 Center for Pharmacogenomics and Individualized Therapy, University of North Carolina at Chapel Hill, Chapel Hill, NC 27599, USA

**Keywords:** rituximab (Rituxan^®^), ofatumumab (Arzerra^®^, Kesimpta^®^), obinutuzumab (Gazyva^®^), CD20 (*MS4A1*), *MKL1* (*MRTFA*), *TGFB1*

## Abstract

Monoclonal antibody (mAb) therapy directed against CD20 is an important tool in the treatment of B cell disorders. However, variable patient response and acquired resistance remain important clinical challenges. To identify genetic factors that may influence sensitivity to treatment, the cytotoxic activity of three CD20 mAbs: rituximab; ofatumumab; and obinutuzumab, were screened in high-throughput assays using 680 ethnically diverse lymphoblastoid cell lines (LCLs) followed by a pharmacogenomic assessment. GWAS analysis identified several novel gene candidates. The most significant SNP, rs58600101, in the gene *MKL1* displayed ethnic stratification, with the variant being significantly more prevalent in the African cohort and resulting in reduced transcript levels as measured by qPCR. Functional validation of *MKL1* by shRNA-mediated knockdown of MKL1 resulted in a more resistant phenotype. Gene expression analysis identified the developmentally associated *TGFB1I1* as the most significant gene associated with sensitivity. qPCR among a panel of sensitive and resistant LCLs revealed immunoglobulin class-switching as well as differences in the expression of B cell activation markers. Flow cytometry showed heterogeneity within some cell lines relative to surface Ig isotype with a shift to more IgG^+^ cells among the resistant lines. Pretreatment with prednisolone could partly reverse the resistant phenotype. Results suggest that the efficacy of anti-CD20 mAb therapy may be influenced by B cell developmental status as well as polymorphism in the *MKL1* gene. A clinical benefit may be achieved by pretreatment with corticosteroids such as prednisolone followed by mAb therapy.

## 1. Introduction

The *MS4A1* gene is a member of the membrane-spanning 4A gene family encoding the B-lymphocyte surface molecule (CD20). Its direct function is largely unknown, but it has been proposed to regulate calcium conductance and be involved in cellular activation [1]. CD20 is specific to developing pre-B, immature, and mature resting (both naïve and memory) B cell lymphocytes [1,2]. It is not expressed in early pro-B cells nor in fully differentiated plasma cells, thus offering specificity for the treatment of B-cell leukemia and lymphomas such as chronic lymphocytic leukemia (CLL), follicular lymphoma, and diffuse large B-cell lymphoma (DLBCL) [3] with limiting off-target toxicity for other cell types. Anti-CD20 antibody therapy has quickly become an important component of treatment regimens for B-cell disorders, with several CD20 mAbs now available. CD20 mAbs can be broadly classified into two categories: type 1 (such as rituximab and ofatumumab, which are capable of binding different epitopes); and type 2 (obinutuzumab), which are distinguished by their ability to induce the translocation of CD20 into lipid rafts within the plasma membrane [4]. Type I antibodies are suggested to be more effective at triggering complement activation due to their induced clustering of CD20 in lipid rafts [5,6,7,8]. It is proposed that this ability to induce translocation may also increase susceptibility to antigen down-regulation [9,10], thus driving the development of type II antibodies such as obinutuzumab. There are at least three proposed cytotoxic mechanisms of action of CD20 mAbs that include antibody-dependent cell-mediated cytotoxicity (ADCC), complement-dependent toxicity (CDC), and direct cell killing. There remains some controversy as to the importance of each; however, CDC was originally considered to be the primary mechanism of cell killing [11]. This study examines the cytotoxicity of these three mAbs, rituximab, ofatumumab, and obinutuzumab, in high-throughput assays under normal media conditions or with human complement present, to more closely mimic physiological conditions and identify the genetic determinants of susceptibility through gene expression and genome-wide association analysis.

The lymphoblastoid cell lines (LCLs) used in this study were obtained from the 1000 Genomes Project [12], which provides a resource to study the effect of human genetic diversity on drug response and disease. As part of a multinational effort, genomes from individuals are available as immortalized CD20^+^ B lymphocytes (LCLs), enabling genome-wide association studies (GWAS) and gene expression studies in large multi-ethnic cohorts. LCLs are thus ideally suited for studying the genetic determinants influencing sensitivity toward anti-CD20 therapies. The LCLs are prepared from peripheral blood B cells of donors immortalized by in vitro transformation with Epstein–Barr virus. Each cell line is thus a population of genetically matched cells, yet significant B cell heterogeneity may exist with respect to immunoglobulin isotypes as well as differences in activation and differentiation pathways [13].

Gene expression analysis suggested differences in B cell developmental status differentiate sensitive from resistant cells. B cell development and maturation is a complex, multi-stage process, generating numerous B cell intermediates and subtypes reflected by a changing repertoire of cell surface markers and functionality. Circulating naïve B cells express surface-bound IgM/D. Cellular activation by antigenic binding through the IgM/BCR (B cell Receptor Complex), as well as cytokine stimulation and interaction with helper T cells, induces immunoglobulin class-switching to IgG/A/E with additional affinity maturation of the immunoglobulin through somatic hypermutation in germinal centers (GC) of secondary lymphoid tissue. GC-derived B cells then differentiate into either relatively quiescent memory B cells, regulatory B cells (B_regs_), or terminally differentiate into more metabolically active antibody-secreting plasma cells. Antigen engagement by the BCR is essential to B cell activation and differentiation, driving the clustering of BCR complexes along with co-receptors into microdomains organized around lipid rafts. Amplification of signaling occurs by the fusion of these microdomains. Fusion is mediated by the cytoskeleton and Src-family kinases to form larger signaling and endocytic regions on the cell surface [14]. CD20 is reported to both localize in lipid rafts [15] as well as interact with the BCR [16] and is suggested to affect signaling by mediating calcium flux [1,17,18]. The BCR also plays an important role in antigen presentation. Antigen bound to the BCR is endocytosed, and processed antigen peptides are then loaded on MHCII, returned to the cell surface, and presented to the helper T cells that participate in B cell germinal center maturation [19,20].

Genome-wide association analysis identified a novel gene, *MKL1*/megakaryoblastic leukemia 1 (also known as *MRTF-A* (myocardin-related transcription factor-A), associated with drug sensitivity. *MKL1* is a transcriptional regulator controlling the expression of cytoskeletal genes. While a role for *MKL1* in hematopoietic development is implicated by its involvement with the t(1;22) translocation specific to acute megakaryoblastic leukemia, there is limited research examining an *MKL1* role during B cell differentiation. B cells from a patient with an intronic deletion in *MKL1* have been recently characterized and shown to have elevated levels of MKL1, which was associated with a number of phenotypic changes: increased actin content; hyperproliferation; genomic instability; decreased expression of CD11a integrin molecules; and decreased homotypic aggregation [21].

## 2. Materials and Methods

### 2.1. Cell Lines and Drugs

This study was performed using 680 lymphoblastoid cell lines (LCLs) from the 1000 Genomes Project [12] after removing first-degree relatives, as previously described [22]. The LCLs selected were derived from nine geographically and ethnically diverse populations: Utah residents with European ancestry (CEU); Han Chinese in Beijing, China (CHB); Japanese in Tokyo, Japan (JPT); Luhya in Webuye, Kenya (LWK); Residents of Los Angeles, California with Mexican ancestry (MXL); Tuscans in Italy (TSI); Yoruban in Ibadan, Nigeria (YRI); British from England and Scotland (GBR); and Colombian in Medellin, Colombia (CLM). The lymphoma lines in this study, DB, Granta-519, HT, and RL, were authenticated by STR analysis (Genetica). Cell lines were cultured at 37 °C and under 5% CO_2_ using RPMI medium 1640 and 15% fetal bovine serum (Sigma, St. Louis, MO, USA).

Rituximab (Rituxan^®^; Genentech, Inc., San Francisco, CA, USA) and ofatumumab (Arzerra^®^; Genmab, Copenhagen, Denmark) were purchased through the North Carolina Cancer Hospital pharmacy. Obinutuzumab (Gazyva^®^; Roche, Basel, Switzerland) was kindly provided by Angela Facul (Universidade Luterana do Brasil, ULBRA).

### 2.2. Drug Response Assays

The cytotoxic drug response of three anti-CD20 mAbs, rituximab, ofatumumab, and obinutuzumab, was compared among 680 lines. LCLs were plated into 384-well plates at approximately 4000 cells/well in a total volume of 50 μL under both normal cell culture conditions (ofatumumab and obinutuzumab) or in the presence of 25% human serum (as a source of complement) and with or without 10 μg/mL of each antibody. In initial screening experiments, which were part of a larger drug screening project [23], pooled human serum (Sigma Aldrich) was used for 72 h, followed by overnight exposure to Alamar Blue (Invitrogen, Waltham, MA, USA) to assess viability. An Infinite F200 microplate reader with Connect Stacker (Tecan US, Chapel Hill, NC, USA) and iControl software (Version 1.6) was used to measure the fluorescence intensity of the viability dye at EX535nm and EM595nm. Viability was expressed relative to samples without antibodies using 10% DMSO samples for background subtraction. Each cell line was assayed twice as quadruplicates. In subsequent experiments, cells were treated for 24 h in the presence of pooled human serum (Innovative Research, Novi, MI, USA), followed by overnight incubation with Alamar Blue.

### 2.3. Gene Expression Analyses

Gene expression analyses were conducted using previously established procedures [23]. RNA-Seq read-count data from the Geuvadis project [24] was available for a subset of 272 individuals in our assays. Extensive quality control was used to remove technical replicates, correct for over-dispersion, and normalize for library depth [25,26]. The correlation between baseline gene expression and cell sensitivity after treatment with rituximab, obinutuzumab, and ofatumumab was assessed by a two-stage multivariate linear regression model specified as follows:

First stage: G_i_ = β_0_ + L_i_*β + ϵ_i_, ϵ_i_ ~ N(0, σ^2^)

Second stage: ***Y_ij_*** = *β*_0_ + *β*_1_*PC1_*i*_ + *β*_2_*PC2_*i*_ + *β*_3_*PC3_*i*_ + *β*_4_*Sex_i_ + *β*_5_*ϵ_i_ + ***e_ij_***, ***e**_ij_* ∼ *N*(**0**,**∑**), where, in the first stage: G_i_ is the quality-controlled RNA-Seq read count of gene *g* for individual *i*, L_i_ is a vector of indicator variables for the lab in which the RNA-Seq was conducted for individual *i*, β is a vector of the regression parameters, and ϵ_i_ are the residuals = observed read count for gene *g*-predicted gene read count for the gene for an individual *I*; in the second stage: Y_ij_ is the vector of normalized responses for the one concentration for the mAb for each individual *i*; the Eigenvalues for the first three principal components, PC_1_, PC_2_, and PC_3_ are calculated using EigenStrat [27]; and *i* is an indicator variable denoting gender.

In the first stage, linear regression is performed using the limma package [28] to remove the lab batch effects. G_i_ is the quality-controlled RNA-Seq read count of gene *g* for individual *i*. L_i_ is a vector indicating the lab in which the RNA-Seq was conducted for an individual. β is a vector of the regression parameters, and ϵ_i_ are the residuals (= observed read count for gene *g*-predicted gene read count for the gene for individual *i*). To minimize extreme residual values from having an excessive influence on parameter estimates, residuals were log-transformed and standardized prior to use in the second stage. In the second stage, linear regression was performed using the R function lm() in package stats v3.4.0 [29] for each gene *g*. Y_ij_ is the vector of normalized responses for the six concentrations of the drug for individual *i*; the Eigenvalues for the first three principal components, PC_1_, PC_2_, and PC_3_, are calculated using EigenStrat [27] with *I* as an indicator variable denoting gender. Significant results from the second stage were obtained after correcting for multiple testing on a per-drug basis using: the Bonferroni correction with a significance level of *p* < 0.05 for the gene expression analysis; and the Benjamini–Hochberg method [30] with a false discovery rate of q < 0.25 for the transcript expression analysis (R function *p.adjust (*https://www.R-project.org/, accessed on 20 September 2019), package stats v3.4.0) [29].

Unsupervised and supervised enrichment analyses using gene set enrichment analysis (GSEA) and gene ontology (GO) analysis were performed using the ClusterProfiler R package [31]. The most statistically significant genes (*p*-value < 0.05) associated with ofatumumab sensitivity were used to identify signaling pathways involved in the differences between LCLs sensitive and resistant to CD20 mAbs treatment.

### 2.4. Genome-Wide Association Studies (GWAS)

Genotype data for the 1000 Genome cell lines from the Illumina HumanOnmi2.5 platform was obtained from the 1000 Genomes database [12] (https://www.internationalgenome.org/data-portal/data-collection/phase-1, accessed on 1 May 2015) and processed as described elsewhere [32]. Briefly, we performed quality control on the genotype data to remove non-autosomal SNPs or SNPs with a call rate <95%, minor allele frequency (MAF) < 0.01, or Hardy-Weinberg equilibrium (HWE) *p*-value < 1 × 10^−6^. After routine quality control, 1,510,701 SNPs were used for GWAS, as described in Abdo et al. [33]. We conducted separate GWAS for rituximab, ofatumumab, and obinutuzumab sensitivity. To test the association between autosomal SNPs and cell viability, we performed linear regression using plink (http://pngu.mgh.harvard.edu/purcell/plink/, accessed on 20 September 2019) under a genotypic model, adjusted for sex, temperature, growth, experimental batch, and the Eigenvalues from the first three principal components using EigenStrat [27] as covariates. The most statistically significant SNP within the gene with the most biological interest was selected for downstream analysis.

### 2.5. MKL1 Knockdown

Stable knockdown cell lines were prepared as previously described [34] by lentiviral transduction using a combination of five individual *MKL1* shRNAs targeting separate regions of the mRNA transcript (Open Biosystems TRC1 library, obtained through the Lenti-shRNA Core Facility University of North Carolina). Control cell lines were prepared using a scrambled shRNA sequence. 1 μg/mL of puromycin was used to select positive cell lines. Knockdown was confirmed by Western blotting.

### 2.6. RNA Isolation and qPCR

Total cellular RNA was extracted from approximately 1 × 10^−6^ cells using Quick-RNA Microprep Kit (Zymo Research, Irvine, CA, USA). Purified RNA was then reverse-transcribed into cDNA using Verso cDNA Synthesis Kit (Thermo Scientific, Waltham, MA, USA). Conventional qPCR was performed using PowerUp SYBR Green Master Mix (Applied Biosystems, Waltham, MA, USA). Reactions were conducted in quadruplicate in 10 μL volumes using 500nM of forward and reverse primers prepared by Sigma Genosys (Millipore Sigma, Burlington, MA, USA) and run on the Applied Biosystems QuantStudio 6 Flex Real-Time PCR System. Primer pairs used are provided (Table S1). Thermocycler parameters were: 50 °C for 2 min, 95 °C for 10 min, and then 40 cycles of 95 °C for 15 s followed by 60 °C for 1 min. The data were analyzed using the delta C_t_ method normalized against *GAPDH*.

### 2.7. Homotypic Aggregation

Homotypic aggregation or cell clump size was estimated from micrographs taken 30 min following the dispersion of cells by pipetting and pulse centrifugation. Images were captured using a Nikon Eclipse Ts2R microscope with a 10x objective and NIS-Elements software. 8-bit grayscale images were converted to binary then particles were defined using the best-fit ellipse selection tool. The particle areas were then estimated with ImageJ software (National Institute of Health) with a size defined as 2000–∞ (inch^2^, analyze particles) within one field of view for each cell line.

### 2.8. Western Blotting

Western blotting was performed as previously described [35], except 5% fish skin gelatin (Sigma) and 2% Bovine Serum Albumin (Thermo Scientific) in Dulbecco’s PBS was used as a blocking buffer. Antibodies included: rabbit αCD20 antibody (Thermo Scientific); rabbit αMKL1 (Bethyl Laboratories, Waltham, MA, USA); and mouse αGAPDH (R&D systems, Minneapolis, MN, USA) was used as load control. Fluor-conjugated secondary antibodies from LiCOR were used as needed. Detection was carried out on a LiCOR Odyssey to visualize immunoreactive bands (LI-COR Biosciences, Lincoln, NE, USA). Protein levels were quantified using NIH Image 1.61.

### 2.9. Flow Cytometry

1.0 × 10^6^ cells were centrifuged, then resuspended in 50 μL cold Dulbecco’s PBS buffer containing 0.1% sodium azide, 1%FBS, and 1% human serum, then kept on ice for 30 min. Cells were washed and resuspended in the same buffer except with 2%FBS as the only serum present. Antibodies used included: αCD20-PE (eBioscience, San Diego, CA, USA); αIgG-FITC (Invitrogen); and αIgM-BV711 (BioLegend San Diego, CA, USA). Antibodies were added to samples for an additional 45 min before adding an equal volume of buffer. Samples were centrifuged, washed, and pellets resuspended in 0.5 mL of the same buffer containing either TOPRO-3 (Invitrogen) or DAPI (Thermo Scientific) as a viability dye. Surface expression analysis was performed on an Attune NxT V6 flow cytometer (Thermo Scientific). Data analysis was performed with FlowJo software (Tree Star, Inc., Ashland, OR, USA). For sorting experiments, 10^7^ cells were stained as above with αIgG except omitting sodium azide from the staining buffer and sorted on a FACSAria III (Becton Dickinson, Franklin Lakes, NJ, USA) within the UNC Flow Cytometry Core Facility.

### 2.10. Ethidium Homodimer-1 (EthD-1) Dye Uptake

Cells were plated into 384 well plates at 2.5 × 10^−5^ cells/well in 25ul media. The reaction was begun by the addition of an equal volume containing 8 μM EthD1 (Molecular Probes), ±20 μg/mL of anti-CD20 mAb, and containing 50% human serum. An Infinite F200 microplate reader (Tecan US) and iControl software (Version 1.6) were used to measure the fluorescence intensity of the dye at EX535nm and EM595nm at timed intervals. The fluorescence of untreated cells was subtracted as background from treated cells. Dye uptake is then presented as a percentage relative to a positive control using cells lysed with 0.1% saponin.

### 2.11. Isolation of Exosomes by Differential Centrifugation

Exosomes were isolated by centrifugation of cell cultures at 250× *g* for 10 min to pellet cells. The supernatant was then centrifuged at 1500× *g* for 10 min to remove cell fragments, including apoptotic bodies. Exosomal material was collected in a subsequent 15,000xg centrifugation for 30 min. In some experiments, 10% (*w*/*v*) PEG-10000 (Sigma) in 0.3M NaCl was added to the 15,000× *g* supernatant overnight at −20 °C then centrifuged at 15,000× *g* for 30′ to confirm quantitative removal of CD20 in the preceding fractions. Pelleted fractions were resuspended in lysis buffer and analyzed by western blotting.

## 3. Results

### 3.1. Lymphoblastoid Cell Line Drug Response Results

The cytotoxic drug response of three anti-CD20 mAbs, rituximab (rtx), ofatumumab (ofat), and obinutuzumab (obin) using a single, saturating concentration of 10 μg /mL, were compared using 680 lymphoblastoid cell lines (LCLs) under both normal cell culture conditions (ofat and obin) as well as in the presence of human serum as a source of complement (rtx, ofat, obin) as part of a larger, drug screening project [23]. Alamar Blue was used to estimate cell survival. Direct cell killing by antibody alone (under standard culture conditions) was compared between type I mAb, ofatumumab, and type II mAb, obinutuzumab. Their profiles were similar, with a median survival of 62% versus 64%, respectively, with a relatively strong correlation in cell response between the two mAbs, *R*^2^ = 0.51 (Table S2, Figure S1). All three CD20 mAbs were screened under conditions of complement-mediated cytotoxicity (CDC). Complete cell killing was observed among some cell lines using either obinutuzumab or ofatumumab. Under the screening conditions in this study, obinutuzumab was most effective, with a median survival of 37% compared to ofatumumab at 60% or rituximab at 64% (Table S2). LCL responses were most similar between rtx and ofat, *R*^2^ = 0.447 or obin and ofat, *R*^2^ = 0.441 (Figure S1). There was the least agreement between rtx and obin, *R*^2^ = 0.237, as reflected in the disparity in median viability between these two drugs as well as the range of response for these two mAbs (Table S2, Figure S1). The minimum viability for the most sensitive cell lines was 0% for obinutuzumab compared to 11% for rituximab (Table S2). Likewise, while all cell lines assayed responded to some degree to obinutuzumab with a maximum viability of 75% in the most resistant lines, some cell lines were resistant to cell kill by rituximab (Table S2). Direct cellular cytotoxicity was not a good predictor for cell response to mAb in the presence of complement. Comparing direct cytotoxicity with complement-mediated cytotoxicity for ofatumumab and obinutuzumab yielded weak correlation coefficients of *R*^2^ = 0.13 and *R*^2^ = 0.19, respectively (Figure S1).

### 3.2. Genome-Wide Association Study (GWAS) Analysis

**Figure 1:**
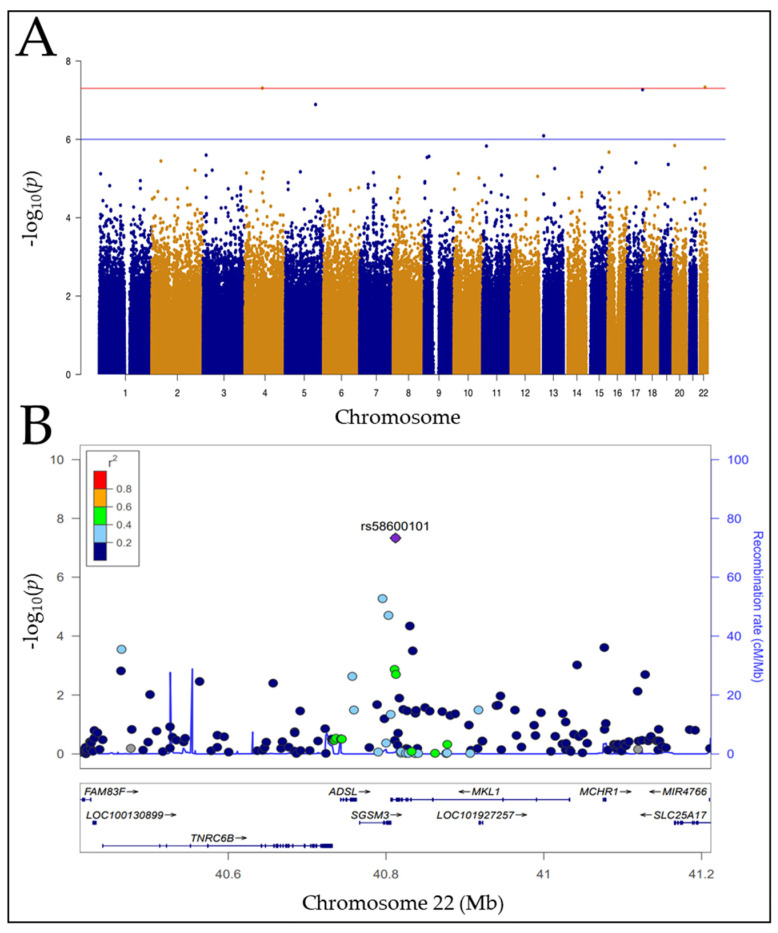
GWAS Manhattan plot and LocusZoom plot for the region with highest significance level. (**A**) Manhattan plot of MAGWAS −log_10_ (*p*-value) over 22 autosomes for the association of genotype and cell viability for ofatumumab +serum. The blue and red lines indicate the thresholds for the genome-wide suggestive significance level of 10^−6^ and the genome-wide significance level of 10^−8^, respectively. (**B**) The LocusZoom plot showing the regional genes surrounding a 0.8 Mb region around the SNP with the highest significance, rs58600101 (purple diamond *p* = 4.654 × 10^−8^). SNPs are color-coded based on their LD r^2^ relative to rs58600101. The fine-scale recombination rates estimated from 1000 Genomes (EUR) data are marked in light blue. In the box below, genes in this region are labeled with horizontal grey arrows indicating the direction of transcription and with rectangles indicating exon regions.

Using genotype data from the 1000 Genomes database, separate GWAS was performed using the response data from the three monoclonal antibodies: rituximab; ofatumumab; and obinutuzumab. Table 1 shows the GWAS results at the suggestive genome-wide significance level or higher (*p*-value < 10^−6^). At the genome-wide suggestive threshold of *p*-value < 10^−6^, there were eight peak associations for ofatumumab and obinutuzumab under normal culture conditions measuring direct cell kill (Figure S2). In the presence of human serum as a complement source, there were a total of seven peak associations for all three antibodies (Figure 1 and Figure S2). At the genome-wide significance level of *p*-value < 5 × 10^−8^, there was one peak association each under normal culture conditions and in the presence of complement. Type II anti-CD20 mAbs, such as obinutuzumab, are reported to have superior direct-killing activity relative to Type I mAbs. Under these conditions of direct cell killing (without human serum as a complement source), the most significant SNPs were associated with obinutuzumab treatment (Table 1). They were rs3904461, an intergenic variant on chromosome 9, and rs77545126 (an intronic variant in the *SCAMP5* gene). An additional SNP, rs60910940 (synonymous variant in *SCAMP5*), was also associated with obinutuzumab treatment.

Under the more physiologic condition with complement present, the most significant association is for SNP, rs58600101, an intronic SNP within the *MKL1* gene with ofatumumab treatment (Table 1). The *MKL1* SNP, rs58600101, exhibits ethnic stratification. The highest MAF of rs58600101 in *MKL1* is 15% in the African cohorts, LWK and YRI compared to 0–1% for all other populations studied (CEU, CHB, JPT, MXL, TSI, GBR, and CLM). *MKL1* was therefore chosen as a candidate gene for functional validation to examine if its expression influenced sensitivity toward anti-CD20 mAbs using shRNA knockdown constructs.

### 3.3. SNP, rs58600101 Affects MKL1 mRNA Abundance

**Figure 2:**
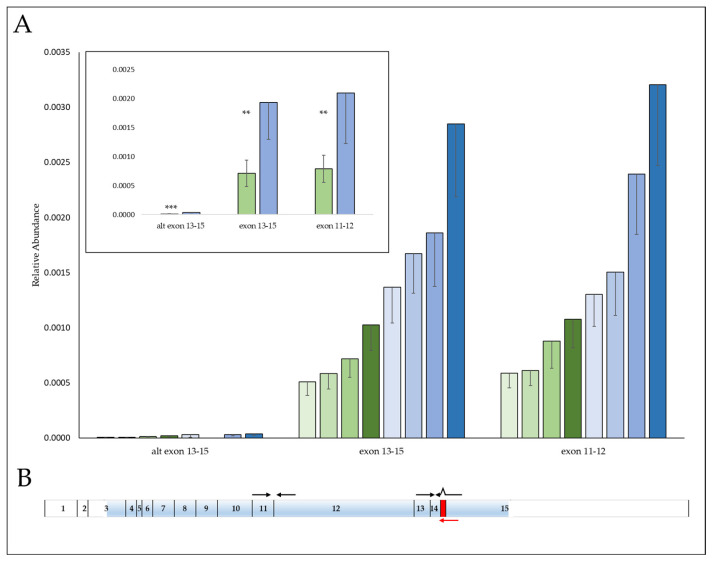
Reduced expression of *MKL1* in SNP rs58600101 variants. (**A**) Comparison of *MKL1* mRNA transcript levels in four homozygous rs58600101 variant (A/A) cell lines (green columns) versus four homozygous wildtype (G/G) cell lines (blue columns). Gene expression levels were measured by qPCR and normalized against *GAPDH*. Results are the average of quadruplicates ± stdev. The inset shows the average ± stdev for each of the two genotype groups. Statistical significance symbols are: **: *p* ≤ 0.01, ***: *p* ≤ 0.001. (**B**) Gene structure of human *MKL1* showing the relative positions of primers used (shown by black arrows). Three primer pairs were used to interrogate *MKL1* mRNA levels by qPCR: MKL1 13–15 (flanking the region of rs58600101); alt MKL1 13–15 (a specific reverse primer shown by the red arrow used to detect an alternate mRNA transcript arising from a splice variant. The resulting 10 bp insertion is indicated by the red block); and MKL1 11–12 (a site distal to rs58600101). Coding regions of the gene are shaded in blue.

SNP, rs58600101, is an intronic SNP located between the last pair of *MKL1* exons 14–15, which help comprise the transcriptional activation domain (TAD) of MKL1 [36]. qPCR was used to compare the gene expression of four homozygous wildtypes against four homozygous variants to determine if this SNP impacts expression. An *MKL1* transcript variant occurs within this region through the use of an alternate splice site, resulting in a frameshift and an early stop codon. For this reason, primers were designed spanning exons 13–15 for both the reference transcript as well as for the splice variant. A third pair of primers were included in a separate region of the gene spanning exons 11–12. *MKL1* gene expression of either of the reference transcripts was reduced in the SNP variants to an average of 0.37 (stdev ± 0.114) relative to wildtype expression (Figure 2). The splice variant was >50-fold less abundant and similar to the reference transcripts; the expression of *MKL1* in the SNP variant averaged 0.34 (stdev ± 0.19) in comparison to wildtype. Therefore, SNP rs58600101 affects transcript levels but did not appear to influence the formation of splice variants.

### 3.4. MKL1 Knockdown Results in a More Resistant Phenotype

**Figure 3:**
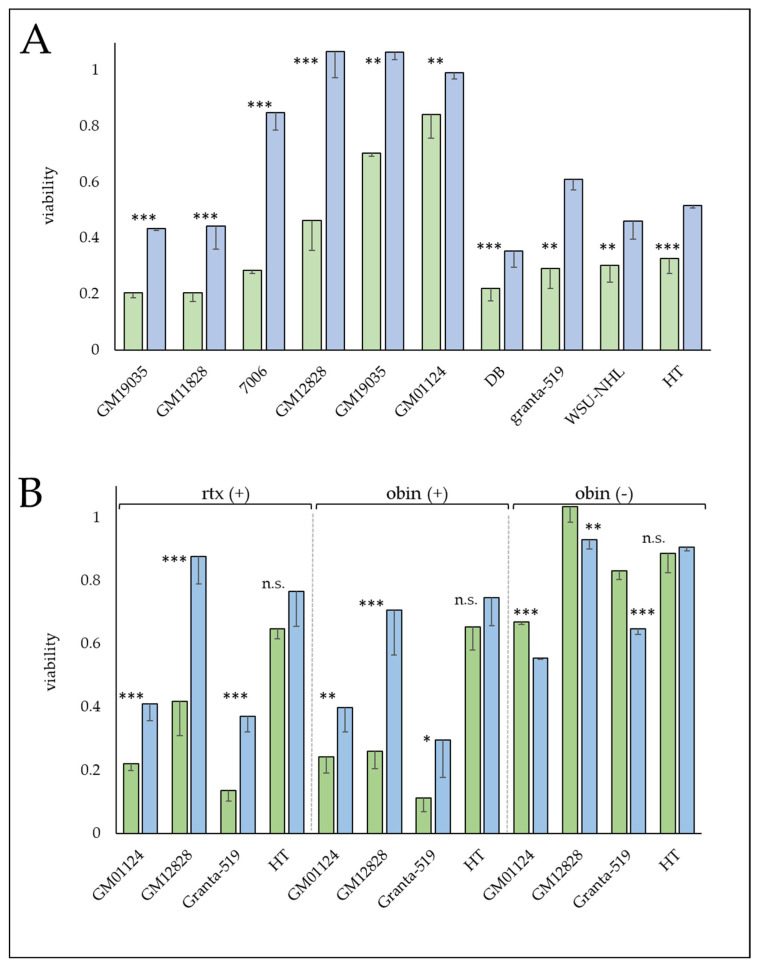
MKL1 knockdown increases resistance toward complement-mediated cytotoxicity by anti-CD20 mAbs. Viability assays of control (empty vector) cell lines (green bars) and corresponding shMKL1 cell lines (blue bars). (**A**) Treatment with ofatumumab in the presence of human serum as a source of complement. (**B**) Viability assay for 2 lymphoblastoid and 2 lymphoma cell lines following treatment with rituximab (rtx) or obinutuzumab (obin) in the presence (+) or absence (−) of human serum as a source of complement. Viability is expressed relative to cells without antibody added (mAb treatment/control) and is the average of triplicates ± stdev. Statistical significance symbols are: ns: *p* > 0.05, *: *p* ≤ 0.05, **: *p* ≤ 0.01, ***: *p* ≤ 0.001.

**Figure 4:**
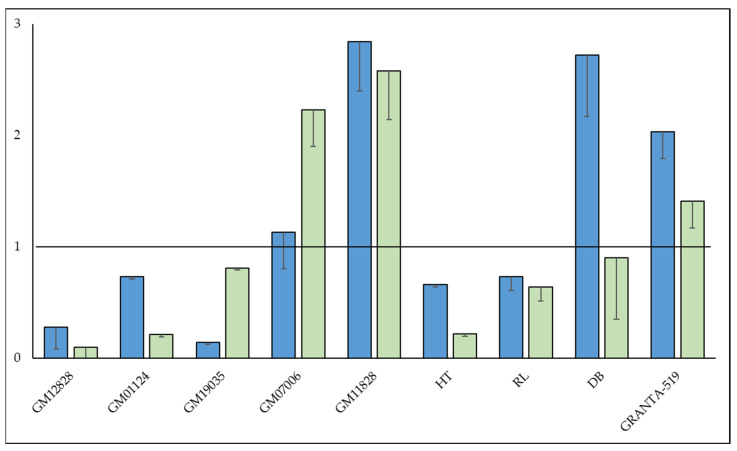
Expression of the target protein, CD20, in MKL1 knockdown cell lines. CD20 expression was measured either as surface expression by flow cytometry (blue bars) or as mRNA abundance by qPCR (green bars). CD20 surface expression is estimated by the mean fluorescent intensity (MFI). Results are expressed as a ratio of shMKL1/control cell line. The results shown are the average of three independent measurements (±stdev). The relative abundance of *MS4A1* (CD20) mRNA (normalized against *GAPDH*) is shown in a likewise manner and is the average of four replicates (± stdev). Values < 1 indicate reduced CD20 in an shMKL1 cell line relative to the control cell line. Values >1 indicate increased expression.

MKL1 protein expression was reduced in multiple LCLs (GM01124, GM07006, GM11828, GM12828, GM19035) and lymphoma lines (DB, Granta-519, HT, RL) using shRNA constructs. Knockdown was confirmed by western blotting to average 87% (stdev ± 9.3%, Figure S3). MKL1 knockdown resulted in a more resistant phenotype in both LCL and lymphoma cell lines in Alamar-CDC assays, with an average increase in resistance by 1.7-fold toward ofatumumab (stdev ± 0.69, Figure 3A). MKL1 knockdown lines appeared cross-resistant to rituximab and obinutuzumab as well when in the presence of complement (Figure 3B). However, MKL1 knockdown lines were not more resistant during direct cell kill by obinutuzumab in the absence of complement. Expression of the target gene, *MS4A1* (*CD20*), was examined by both qPCR and flow cytometry in these cell lines (Figure 4). Reduced *CD20* levels were observed in the majority of sh*MKL1* cell lines as measured by qPCR, with corresponding decreases in CD20 surface expression as well. While all sh*MKL1* cell lines exhibited increased resistance toward mAb treatment, reduced CD20 levels were not seen in four (GM07006, GM11828, DB, Granta-519) of the nine cell lines, suggesting *MKL1* is responsible for additional mechanisms of resistance. Further characterization of the MKL1 knockdown cells (Figure S3) did not show any consistent trends in affecting the growth rates of cells. MKL1 knockdown did not offer increased resistance toward other chemotherapeutic drugs tested (mitomycin C, doxorubicin, vincristine, prednisolone, arsenic trioxide, and 5-fluorouracil) with the possible exception of arsenic trioxide (Figure S3).

### 3.5. Gene Expression Analyses Results

Associations between basal gene expression and complement-mediated cytotoxic drug response were examined for these three anti-CD20 mAbs using publicly available expression data [24]. The gene expression differences shown are significant results following a Bonferroni correction with a significance level of *p* < 0.05 (Figure S6). The *MS4A1* gene, encoding CD20 protein, is the target of anti-CD20 antibodies and is thus an obvious candidate to interrogate for differences in expression between sensitive and resistant cell lines. However, no statistical differences were observed in this analysis. Among the three antibodies, ofatumumab generated the most statistically significant results, with 221 genes identified (*p* < 0.05). Transforming Growth Factor Beta 1 Induced Transcript 1 (*TGF1I1*, *p* < 0.0003) was the most significant gene correlating with sensitivity. Two genes were identified for rituximab (*KCNA6*, *ZNF629*) that were likewise significant for ofatumumab. Eight genes were identified for obinutuzumab (*AIG1*, *WWTR1*, *RP11-545E17.3*, *RP11-251G23.2*, *GAN, IL1RAP*, *RSAD1i* and *PDCD4*). Five of these genes were likewise significantly associated with ofatumumab. The three remaining genes could thus be uniquely assigned to the type II antibody, obinutuzumab, and included: *RSAD1* (*p* < 0.02), *RP11-251G23.2* (*p* < 0.02), and *PDCD4* (*p* < 0.04).

The gene list generated by ofatumumab was examined en masse to identify biological processes that might distinguish sensitive from resistant cells using GSEA (Gene Set Enrichment Analysis) and GO (Gene ontology) enrichment analyses (Figure S4). The GO-enriched annotations identified genes belonging to biological processes involved in ‘vesicle organization’ and ‘positive regulation of cell projection regulation’. The most enriched GSEA annotations were those relating to ribosomal/translational regulation; protein localization, particularly to the endoplasmic reticulum; cytoskeletal organization; nuclear division; and recombinational repair. These results were suggestive of a shift toward a more activated B cell phenotype in the resistant group. Differences impacting the plasma membrane such as those involving cytoskeletal and vesicle organization could also suggest potential repair mechanisms. Supervised gene expression was also undertaken with a gene list that included developmentally associated genes but also the inclusion of genes that could be involved in potential resistance mechanisms (Table S4). Supervised statistical analysis notably revealed that increased expression of *MHCII*-associated genes was associated with the resistant phenotype. *SCAMP5* again appeared as a significant gene during this analysis. Its interaction with the cytoskeleton at the inner plasma membrane surface and involvement in calcium-triggered vesicle fusion followed by exocytosis likewise suggest potential protective mechanisms.

### 3.6. Resistant Cell Lines Exhibit a More Activated B Cell Phenotype

**Figure 5:**
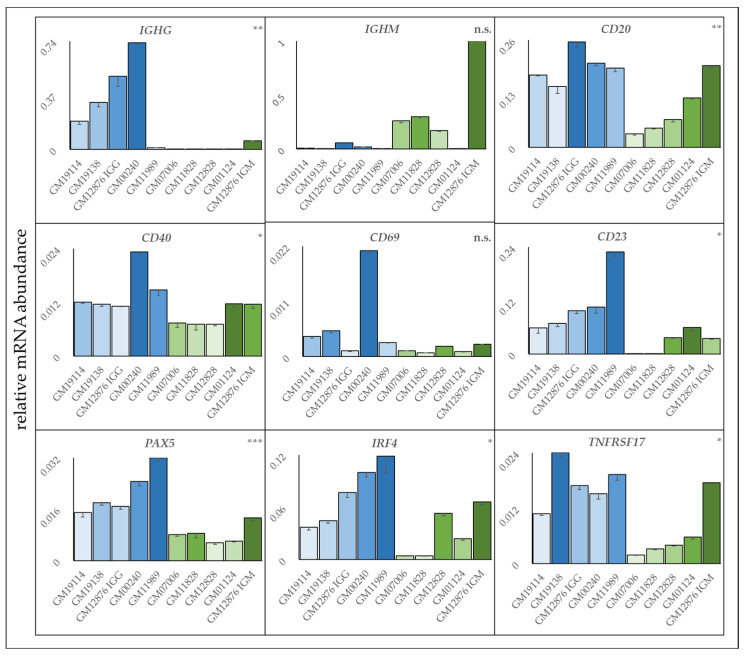
Differences in gene expression between sensitive and resistant LCLs. Gene expression levels were measured by qPCR and normalized against *GAPDH* for four sensitive (GM01124, GM07006, GM11828, GM12828) and four resistant (GM00240, GM19114, GM19138, GM11989) LCLs, as well as an LCL, GM12876, whose mixed population of IgG+ (resistant) and IgM+ (sensitive) expressing cells were separated by FACS. Sensitive cell lines are shown in green. Resistant lines are shown in blue. Results are the average of quadruplicates ± stdev. Statistical significance symbols are ns: *p* > 0.05, *: *p* ≤ 0.05, **: *p* ≤ 0.01, ***: *p* ≤ 0.001.

During the screening of LCLs in viability assays, increased homotypic aggregation was often observed in resistant cell lines relative to more sensitive lines. Therefore, the size of cell aggregate was measured in seven resistant and seven sensitive cell lines. The relative aggregate size of resistant cell lines averaged 778 μm (±101 sem) compared to 161μm (±45.8 sem) for sensitive cell lines (Figure S5, *p* ≤ 0.001). Since homotypic aggregation can be indicative of a more activated B cell, frequently as a consequence of CD40 signaling [37,38], follow-up experiments were then conducted to look for additional evidence of a shift in development or activation status between sensitive and resistant cell lines. Four LCLs identified as sensitive based on the average viability toward all three mAbs and four LCLs identified as resistant were selected and assayed by qPCR for expression of B cell differentiation and activation markers (Figure 5). Several differences were evident in a comparison of sensitive versus resistant cell lines. Most notably, there was evidence of isotype class-switching, with the sensitive group expressing more *IgM* relative to *IgG*. In contrast, resistant cell lines expressed more *IgG* as well as markers associated with an activated B cell phenotype, such as *IRF4*, *TNFSRF17*, *CD40*, and *CD23* (*FcεRII*) [39,40,41,42]. The most significant predictor of resistance was *PAX5* (*p* ≤ 0.0003).

Surface expression of IgG and IgM were then analyzed in 9 LCLs by flow cytometry. As expected, based on results from qPCR data, resistant lines were predominantly IgG^+^, and generally, sensitive cells were more IgM^+^. Some cell lines exhibited mixed populations of both IgG^+^ and IgM^+^ cells (Figure S6). One such cell line was therefore sorted using flow cytometry to determine if, within a genetically matched population of cells (from a single individual), isotype expression alone could be correlated with sensitivity to antibody treatment. The cell line GM12876 was sorted based on IgG expression into two populations: IgG^−^ and IgG^+^. The IgG^−^ population was confirmed by qPCR to be enriched for *IGHM* mRNA transcripts, and conversely, the IgG^+^ population was enriched for *IGHG* (Figure S7). The two sub-populations were assayed for sensitivity toward complement-mediated cell killing by two anti-CD20 mAbs. The IgG^+^ population was more resistant toward antibody treatment. Viability was 237% (*p* ≤ 0.001) higher in IgG^+^ cells relative to the IgM^+^ population when assayed against rituximab (Figure S7). The difference was not as pronounced when using ofatumumab. Viability was 120% (*p* ≤ 0.01) higher in the IgG^+^ population when compared to IgM^+^ cells.

### 3.7. The Glucocorticoid, Prednisolone Increases Sensitivity to CD20 mAbs

Given that there appeared to be an association between mAb resistance and expression of activated B cell markers, a panel of B cell lines was pre-treated with the immunosuppressant, prednisolone for 10 days to essentially deactivate and thus sensitize the cell lines toward CD20 mAb treatment. While not all cell lines appeared responsive to prednisolone, the majority of cells were sensitized. Cell viability was reduced an additional 63% (±0.21 stdev) on average relative to cells not pre-treated with prednisolone following exposure to ofatumumab (+complement, Figure S8). The effect of prednisolone on CD20 surface expression was then examined by flow cytometry. A time course of prednisolone treatment revealed an increase in expression of the drug target, CD20, from 2.5 to 10 days, with no further increase observed at the 20-day time point (Figure S9A,B). CD20 surface expression was also examined following exposure to ofatumumab in both untreated and pre-treated cells (Figure S9C). There was a dramatic 95% (±0.11 stdev) loss of CD20 as estimated by the mean fluorescence intensity (MFI) following a 30 min exposure to ofatumumab (+complement) and is consistent with previous reports noting a loss of CD20 following mAb treatment [9,43,44]. The loss was temporary. CD20 surface expression could be restored to near original levels when tested following the removal of the drug and a 14-day incubation under normal culture conditions. Recovery was thus 77% and 86% (respectively) for the two lymphoblastoid cell lines, GM12876 and GM19189. Unfortunately, prednisolone pretreatment could not prevent loss of CD20 surface expression following mAb treatment (Figure S9D). The loss was proportionately the same in prednisolone-treated cells (94% ± 0.21 stdev). However, due to the overall increase in total CD20 levels, more of the drug target, CD20, was present on the cell surface following mAb drug treatment in prednisolone-treated cultures.

### 3.8. Protective Mechanisms Affecting Complement-Mediated Anti-CD20 mAb Therapy

Identification of *MKL1* and *SCAMP5* suggested differences in cytoskeletal or membrane dynamics may be affecting sensitivity. Potential mechanisms of resistance were examined, including loss of the drug target through either internalization or shedding of CD20 from the cell surface as well membrane repair following complement-mediated pore formation (Figure S10). To examine internalization, LCLs were prelabelled with PE-conjugated anti-CD20 antibody binding to the rituximab epitope. Cells were then treated with ofatumumab binding a distinct epitope along with human serum to initiate complement fixation. After 30 min, cells were processed for flow cytometry. The majority of labeled CD20 (>95%) was lost following treatment (Figure S10A). Internalization of CD20 thus seemed unlikely to account for the loss of CD20 from the cell surface. To examine loss of CD20 through an exocytic mechanism, differential centrifugation followed by western blotting of recovered fractions was used to examine the shedding of CD20 from cells. Results indicate that CD20 is constitutively released from cells. Supernatant from an overnight culture yields an almost equivalent amount of CD20 compared to the cell-associated CD20. The release is also further stimulated by anti-CD20 mAb treatment. A panel of sensitive and resistant cells was compared for the induced release of CD20. The results were suggestive but not conclusive that resistant cells may release more CD20 following mAb treatment compared to a panel of sensitive cell lines (Figure S10B). The strongest results obtained were from EthD1 uptake experiments which were used to examine complement pore formation. Ethidium homodimer-1 (EthD1) is a cell-impermeant dye whose fluorescence increases 40-fold when bound to DNA. We observed that there was similar uptake kinetics between ofatumumab and obinutuzumab, with uptake plateauing at ~10 min following mAb treatment and reaching a maximum of ~20% relative to the positive control. Reduced effects were seen with rituximab. Dye uptake did not plateau until ~30 min and at only 8% relative to the positive control (Figure S10C). These results were consistent with results from the viability data collected. Reduced uptake was observed in resistant cells relative to sensitive cells. These results suggest that while the drug target, CD20, is being shed from the cell surface, it is the kinetics of membrane repair following complement fixation that distinguishes sensitive from resistant cells.

## 4. Discussion

The goal of this study was to contribute to the emerging field of personalized medicine, utilizing an individual’s unique genetic profile to help guide treatment. Our strategy included gene expression analysis and genome-wide association studies (GWAS) following high-throughput in vitro screening of LCLs as an unbiased approach to identify genes affecting sensitivity toward anti-CD20 mAb therapy without reliance on a priori knowledge for potential gene candidates.

GWAS identified the SNP, rs58600101, in *MKL1*, also referred to as Myocardin-related transcription factor A (*MRTFA*), as a significant determinant of sensitivity. MKL1 is a transcriptional co-regulator along with serum response factor (SRF) controlling the expression of cytoskeleton-related genes, including actin [45,46]. MKL1 is itself regulated by RhoA-actin cytoskeletal dynamics triggered by stimuli such as serum, cytokines, or mechanical forces [47,48,49]. The MKL/SRF complex plays a role during B lymphocyte development and has been shown to be a key regulator of TGF-β1 signaling [50,51,52,53]. Knockout of SRF has been shown to lead to a loss of marginal zone B cells as well as reduced expression of IgM, CD19, and the chemokine receptor 4 in B lymphocytes [54]. The *MKL1* SNP, rs58600101, is an intronic SNP located in the 3′ end of the gene between the last pair of exons 14/15. The SNP variant was associated with increased resistance toward anti-CD20 mAbs during high-throughput screening. The variant could be associated with reduced *MKL1* mRNA expression. This was consistent with subsequent shRNA knockdown experiments in which reduced MKL1 expression was accompanied by a more resistant phenotype in both lymphoblastoid as well as lymphoma cell lines. This SNP exhibited ethnic stratification with the minor allele found predominantly in the African population cohort (MAF = 0.170), with only one variant found within the non-African cohort of the 680 cell lines in this study sample. This result suggests that the efficacy of anti-CD20 mAbs may be diminished in patients of African descent.

Gene expression results indicate that differences in B cell development or activation status likely affect sensitivity with potential involvement of the TGF-beta 1 signaling pathway. TGFb1 is an important regulator of B cell development, having been shown to interfere with the expression of cell surface λ light chains during in vitro differentiation of normal human pre-B cells [55] and to inhibit class switching as well as immunoglobulin synthesis [56,57]. Gene expression analysis identified its gene target, *TGFB1l1*, as one of the most significant determinants of sensitivity. TGFB1l1 is a member of the paxillin superfamily of focal adhesion adaptor proteins [58,59] that coordinate multiple protein–protein interactions at the focal adhesion complex and, as such, establishes a physical linkage between extracellular signaling proteins and the cell’s actin cytoskeleton. TGFB1I1 has a dual role, and within the nucleus, it functions as a nuclear receptor coactivator regulating receptor transcriptional activity of glucocorticoid, androgen, mineralocorticoid, and progesterone receptors [60].

A previous, smaller study by this group relying on linkage analysis of pedigreed cell lines identified a similar gene, *CBLB*, as a determinant of anti-CD20 sensitivity. Knockdown experiments resulted in increased CD20 clustering on the cell surface and increased resistance toward anti-CD20 mAbs [34]. Like TGFB1I1, CBLB is an adaptor protein operating at the plasma membrane that is reported to be a negative immune regulator [61,62]. There may be a direct interaction with TGFB1I1 and CBLB. Ryan et al. [63] characterized the more tissue-restricted CBL isoform, CBLC. They reported that the RING finger domain of CBLC, which is shared with its isoform CBLB, binds to the LIM-2 domain of TGFB1I1, enhancing the ubiquitin ligase activity of CBLC. In addition, CBLB is reported to mediate TGF-beta sensitivity in primary T cells [64]. Together, the identification of TGFB1l1, MKL1, and CBLB in these studies suggest that developmentally driven, juxtamembrane organizational changes of the cytoskeleton are affecting sensitivity toward anti-CD20 mAbs perhaps by influencing plasma membrane repair processes following an injury during complement-mediated pore formation.

The developmental/activation status of LCLs was examined more closely for evidence of immunoglobulin class-switching among a group of sensitive versus resistant cell lines by flow cytometry. Sensitive lines were predominantly IgM^+^, while resistant lines were enriched in class-switched IgG^+^ cells. Other cell lines used in this study were observed to have a more evenly mixed population of IgM^+^ and IgG^+^ cells. While this Ig isotype-mixed population of cells within a cell line may interfere with the results obtained during gene expression analysis, it allowed for the separation of a single cell line, one individual, based on the surface expression of the Ig isotype. The cell line GM12876 was thus separated by flow cytometry based on Ig surface expression. Consistent with the results above, the IgG^+^ subline was more resistant toward anti-CD20 mAb treatment relative to the IgM^+^ subline.

IgG-switched cells can be associated with a more activated status. In mice, IgG memory cells have been shown to be more likely to differentiate into antibody-secreting plasma cells following re-stimulation with antigen compared to IgM memory cells [65,66]. Membrane immunoglobulin (mIg) isotypes, mIgM and mIgG, differ in their transmembrane, cytoplasmic tails; mIgM has only three amino acids, whereas mIgG has a highly species-conserved 28 amino acids that likely result in important signaling differences [67]. Experiments using chimeric IgMG receptors containing the longer IgG tail have shown increased calcium flux, enhanced plasma cell differentiation, and heightened antibody secretion [68,69,70]. Immunoglobulin isotype may also confer distinct mechanical properties affecting plasma membrane function. Surface expression of IgG is associated with greater traction forces when encountering antigens, with actin remodeling contributing to traction force generation and the requirement of signaling molecules (Lyn, Syk, Btk, and Vav3) as well as adapter molecules (Grb2, Cbl, Dok3) for sustained activation of these forces [71,72]. Thus, a proposed model to account for membrane immunoglobulin isotype directly affecting sensitivity would be: anti-CD20 mAb (rituximab, ofatumumab) binding to CD20 induces translocation [4,5,6,7,8,15,16] to the BCR and subsequent complement fixation and pore formation induces calcium influx [1,17,18]. The influx of calcium localized at the BCR may mimic the process of antigen binding to mIg, which likewise triggers calcium flux followed by actin remodeling and endocytosis of the bound antigen for subsequent antigen processing. Endocytosis could result in the removal of mIg as well as CD20 and damaged membrane from the cell surface. This process would be more pronounced in IgG^+^ cells [69,70]. However, since the calcium-regulated exocytic vesicle protein SCAMP5 appeared in both supervised gene expression and GWAS analyses in this study, a protective exocytic mechanism cannot be excluded.

Differences in gene expression, as well as differences in homotypic aggregation between resistant and sensitive cells, suggest that B cell activation status affects sensitivity to anti-CD20 mAbs. Based on this hypothesis, the glucocorticoid prednisolone was used as an immunosuppressant to deactivate and thus sensitize resistant cell lines in this study. Prednisone is presently used concurrently as a component of the cancer drug combination R-CHOP (Rituximab- cyclophosphamide, doxorubicin hydrochloride, vincristine (Oncovin), prednisone) for treatment against B cell malignancies. This combination regimen is typically used for leukemia and lymphomas. Rituximab treatment may also be used for a variety of autoimmune conditions such as rheumatoid arthritis, myasthenia gravis, multiple sclerosis, antiphospholipid syndrome, and potentially, systemic lupus erythematosus (for reviews, see: Refs. [73,74,75]). Prednisolone pretreatment resulted in sensitization of the treated cells and was accompanied by continual increases in CD20 surface expression for up to 10 days suggesting an advantage in a sequential regimen of prednisone followed by anti-CD20 mAb therapy such as with rituximab.

## 5. Conclusions

The purpose of this study was to pharmacogenomically assess several hundred ethnically diverse lymphoblastoid cell lines for response to anti-CD20 mAb therapy to identify genes affecting sensitivity or having prognostic value. Results provided several insights. Multiple gene candidates were identified with a focus on the *MKL1* gene, a transcriptional activator controlling the expression of cytoskeletal genes. A polymorphism in *MKL1* was associated with mAb resistance and exhibited ethnic stratification suggesting the efficacy of anti-CD20 mAb therapy may be impacted by ethnicity. In addition, results suggested that B cell developmental/activation status likewise influences sensitivity toward anti-CD20 mAb therapy, with some increase in sensitivity occurring following extended pretreatment with the immunosuppressant prednisolone. Exploring potential protective mechanisms toward complement-mediated mAb therapy suggests that while CD20 is constitutively shed from cells, it is the kinetics of membrane repair following complement fixation that distinguishes sensitive from resistant cells.

## Figures and Tables

**Table 1:** GWAS summary of SNPs significantly associated with drug response. SNPs associated with anti-CD20 mAbs under normal culture conditions (“−“, without complement) and in the presence of human serum (“+”, with complement) at the genome-wide suggestive significance level of *p*-value < 10^−6^ or higher are shown for the 3 mAbs. The results are sorted by presence or absence of complement and *p*-value. Variant consequence obtained from Ensembl VEP (Ensembl GRCh38 release 108,15October 2022). Chr: Chromosome, SNP: Single Nucleotide Polymorphism.

Complement Present	Drug (mAb)	Chr	SNP	*p*-Value	Consequence	Host Gene Symbol	Host Gene Ensembl ID
+	ofatumumab	22	rs58600101	4.654 × 10^−8^	intron_variant, regulatory_region_variant	MKL1 (MRTFA)	ENSG00000196588
+	ofatumumab	17	rs115172145	5.369 × 10^−8^	intron_variant	PRPSAP1	ENSG00000161542
+	rituximab	13	rs9542996	1.272 × 10^−7^	intergenic_variant	-	-
+	ofatumumab	5	rs111502792	1.291 × 10^−7^	intron_variant	KCTD16	ENSG00000183775
+	obinutuzumab	13	rs9540041	1.797 × 10^−7^	intergenic_variant	-	-
+	ofatumumab	13	rs114465368	8.115 × 10^−7^	intergenic_variant	-	-
+	ofatumumab	13	rs116833413	8.125 × 10^−7^	intergenic_variant	-	-
−	obinutuzumab	9	rs3904461	1.174 × 10^−8^	intergenic_variant	-	-
−	obinutuzumab	15	rs77545126	3.218 × 10^−7^	intron_variant	SCAMP5	ENSG00000198794
−	obinutuzumab	14	rs75671053	3.254 × 10^−7^	intron_variant	AKAP6	ENSG00000151320
−	obinutuzumab	4	rs58361570	3.516 × 10^−7^	intergenic_variant	-	-
−	obinutuzumab	15	rs60910940	3.582 × 10^−7^	synonymous_variant	SCAMP5	ENSG00000198794
−	ofatumumab	7	rs79213207	7.742 × 10^−7^	intergenic_variant	-	-
−	obinutuzumab	13	rs80101498	7.908 × 10^−7^	intergenic_variant	-	-
−	obinutuzumab	11	rs6579002	8.263 × 10^−7^	intergenic_variant	-	-

## Data Availability

The material generated or analyzed during this study are included within the article and its supplementary information files except for the sequence of the 1000Genomes cell lines which is available from the European Bioinformatics Institute at the following link: http://ftp.1000genomes.ebi.ac.uk/vol1/ftp/ and RNA-Seq read-count data from the Geuvadis project [24].

## Supplementary Materials

The following supporting information can be downloaded at: https://www.mdpi.com/article/10.3390/cells12121574/s1, Table S1: List of primers used for qPCR. Table S2: Summary of viability results. Figure S1: Scatterplots of viability data for rituximab, ofatumumab, and obinutuzumab. Figure S2: Manhattan plots. Figure S3: Characterization of MKL1 knockdown. Table S3: Gene expression levels that are significantly correlated with sensitivity. Figure S4: Gene ontology (GO) and gene set enrichment analysis (GSEA). Table S4: supervised gene expression list. Table S5: Supervised gene expression analysis results. Figure S5: Increased homotypic aggregation in resistant cells. Figure S6: Surface immunoglobulin isotype expression correlates with sensitivity. Figure S7: Separation of a lymphoblastoid cell line based on Ig isotype results in subpopulations with different sensitivity to anti-CD20 mAbs. Figure S8: Prednisolone pretreatment increases sensitivity toward anti-CD20 mAb. Figure S9: CD20 surface expression following prednisolone and anti-CD20 mAb treatment. Figure S10: Potential protective mechanisms: endocytosis; excision; and membrane repair.

## References

[1] Tedder T.F., Engel P. (1994). CD20: A regulator of cell-cycle progression of B lymphocytes. Immunol. Today.

[2] Stashenko P., Nadler L.M., Hardy R., Schlossman S.F. (1980). Characterization of a human B lymphocyte-specific antigen. J. Immunol..

[3] Salles G., Barrett M., Foa R., Maurer J., O’Brien S., Valente N., Wenger M., Maloney D.G. (2017). Rituximab in B-Cell Hematologic Malignancies: A Review of 20 Years of Clinical Experience. Adv. Ther..

[4] Deans J.P., Robbins S.M., Polyak M.J., Savage J.A. (1998). Rapid redistribution of CD20 to a low density detergent-insoluble membrane compartment. J. Biol. Chem..

[5] Chan H.T., Hughes D., French R.R., Tutt A.L., Walshe C.A., Teeling J.L., Glennie M.J., Cragg M.S. (2003). CD20-induced lymphoma cell death is independent of both caspases and its redistribution into triton X-100 insoluble membrane rafts. Cancer Res..

[6] Cragg M.S., Morgan S.M., Chan H.T., Morgan B.P., Filatov A.V., Johnson P.W., French R.R., Glennie M.J. (2003). Complement-mediated lysis by anti-CD20 mAb correlates with segregation into lipid rafts. Blood.

[7] Cragg M.S., Glennie M.J. (2004). Antibody specificity controls in vivo effector mechanisms of anti-CD20 reagents. Blood.

[8] Teeling J.L., French R.R., Cragg M.S., van den Brakel J., Pluyter M., Huang H., Chan C., Parren P.W., Hack C.E., Dechant M. (2004). Characterization of new human CD20 monoclonal antibodies with potent cytolytic activity against non-Hodgkin lymphomas. Blood.

[9] Beers S.A., French R.R., Chan H.T., Lim S.H., Jarrett T.C., Vidal R.M., Wijayaweera S.S., Dixon S.V., Kim H., Cox K.L. (2010). Antigenic modulation limits the efficacy of anti-CD20 antibodies: Implications for antibody selection. Blood.

[10] Herter S., Herting F., Mundigl O., Waldhauer I., Weinzierl T., Fauti T., Muth G., Ziegler-Landesberger D., Van Puijenbroek E., Lang S. (2013). Preclinical activity of the type II CD20 antibody GA101 (obinutuzumab) compared with rituximab and ofatumumab in vitro and in xenograft models. Mol. Cancer Ther..

[11] Reff M.E., Carner K., Chambers K.S., Chinn P.C., Leonard J.E., Raab R., Newman R.A., Hanna N., Anderson D.R. (1994). Depletion of B cells in vivo by a chimeric mouse human monoclonal antibody to CD20. Blood.

[12] Genomes Project C., Abecasis G.R., Auton A., Brooks L.D., DePristo M.A., Durbin R.M., Handsaker R.E., Kang H.M., Marth G.T., McVean G.A. (2012). An integrated map of genetic variation from 1,092 human genomes. Nature.

[13] SoRelle E.D., Dai J., Bonglack E.N., Heckenberg E.M., Zhou J.Y., Giamberardino S.N., Bailey J.A., Gregory S.G., Chan C., Luftig M.A. (2021). Single-cell RNA-seq reveals transcriptomic heterogeneity mediated by host-pathogen dynamics in lymphoblastoid cell lines. Elife.

[14] Pierce S.K. (2002). Lipid rafts and B-cell activation. Nat. Rev. Immunol..

[15] Petrie R.J., Deans J.P. (2002). Colocalization of the B cell receptor and CD20 followed by activation-dependent dissociation in distinct lipid rafts. J. Immunol..

[16] Polyak M.J., Li H., Shariat N., Deans J.P. (2008). CD20 homo-oligomers physically associate with the B cell antigen receptor. Dissociation upon receptor engagement and recruitment of phosphoproteins and calmodulin-binding proteins. J. Biol. Chem..

[17] Bubien J.K., Zhou L.J., Bell P.D., Frizzell R.A., Tedder T.F. (1993). Transfection of the CD20 cell surface molecule into ectopic cell types generates a Ca2+ conductance found constitutively in B lymphocytes. J. Cell Biol..

[18] Li H., Ayer L.M., Lytton J., Deans J.P. (2003). Store-operated cation entry mediated by CD20 in membrane rafts. J. Biol. Chem..

[19] Merkenschlager J., Eksmond U., Danelli L., Attig J., Young G.R., Nowosad C., Tolar P., Kassiotis G. (2019). MHC class II cell-autonomously regulates self-renewal and differentiation of normal and malignant B cells. Blood.

[20] Shimoda M., Li T., Pihkala J.P., Koni P.A. (2006). Role of MHC class II on memory B cells in post-germinal center B cell homeostasis and memory response. J. Immunol..

[21] Record J., Sendel A., Kritikou J.S., Kuznetsov N.V., Brauner H., He M., Nagy N., Oliveira M.M.S., Griseti E., Haase C.B. (2020). An intronic deletion in megakaryoblastic leukemia 1 is associated with hyperproliferation of B cells in triplets with Hodgkin lymphoma. Haematologica.

[22] Peters E.J., Motsinger-Reif A., Havener T.M., Everitt L., Hardison N.E., Watson V.G., Wagner M., Richards K.L., Province M.A., McLeod H.L. (2011). Pharmacogenomic characterization of US FDA-approved cytotoxic drugs. Pharmacogenomics.

[23] Akhtari F.S., Green A.J., Small G.W., Havener T.M., House J.S., Roell K.R., Reif D.M., McLeod H.L., Wiltshire T., Motsinger-Reif A.A. (2021). High-throughput screening and genome-wide analyses of 44 anticancer drugs in the 1000 Genomes cell lines reveals an association of the NQO1 gene with the response of multiple anticancer drugs. PLoS Genet..

[24] Lappalainen T., Sammeth M., Friedlander M.R., ‘t Hoen P.A., Monlong J., Rivas M.A., Gonzalez-Porta M., Kurbatova N., Griebel T., Ferreira P.G. (2013). Transcriptome and genome sequencing uncovers functional variation in humans. Nature.

[25] Robinson M.D., McCarthy D.J., Smyth G.K. (2010). edgeR: A Bioconductor package for differential expression analysis of digital gene expression data. Bioinformatics.

[26] McCarthy D.J., Chen Y., Smyth G.K. (2012). Differential expression analysis of multifactor RNA-Seq experiments with respect to biological variation. Nucleic Acids Res..

[27] Price A.L., Patterson N.J., Plenge R.M., Weinblatt M.E., Shadick N.A., Reich D. (2006). Principal components analysis corrects for stratification in genome-wide association studies. Nat. Genet..

[28] Ritchie M.E., Phipson B., Wu D., Hu Y., Law C.W., Shi W., Smyth G.K. (2015). limma powers differential expression analyses for RNA-sequencing and microarray studies. Nucleic Acids Res..

[29] Team R.C. R: A Language and Environment for Statistical Computing. https://www.r-project.org/.

[30] Benjamini Y., Hochberg Y. (1995). Controlling the false discovery rate: A practical and powerful approach to multiple testing. J. R. Stat. Soc. Ser. B Methodol..

[31] Yu G., Wang L.G., Han Y., He Q.Y. (2012). clusterProfiler: An R package for comparing biological themes among gene clusters. OMICS.

[32] Brown C.C., Havener T.M., Medina M.W., Jack J.R., Krauss R.M., McLeod H.L., Motsinger-Reif A.A. (2014). Genome-wide association and pharmacological profiling of 29 anticancer agents using lymphoblastoid cell lines. Pharmacogenomics.

[33] Abdo N., Xia M., Brown C.C., Kosyk O., Huang R., Sakamuru S., Zhou Y.H., Jack J.R., Gallins P., Xia K. (2015). Population-based in vitro hazard and concentration-response assessment of chemicals: The 1000 genomes high-throughput screening study. Environ. Health Perspect..

[34] Jack J., Small G.W., Brown C.C., Havener T.M., McLeod H.L., Motsinger-Reif A.A., Richards K.L. (2018). Gene expression and linkage analysis implicate CBLB as a mediator of rituximab resistance. Pharm. J..

[35] Small G.W., McLeod H.L., Richards K.L. (2013). Analysis of innate and acquired resistance to anti-CD20 antibodies in malignant and nonmalignant B cells. PeerJ.

[36] Reed F., Larsuel S.T., Mayday M.Y., Scanlon V., Krause D.S. (2021). MRTFA: A critical protein in normal and malignant hematopoiesis and beyond. J. Biol. Chem..

[37] Cao L., Yoshino T., Nishiuchi R., Yamadori I., Akagi T. (1995). Homotypic cell aggregation via conformational change of CD44 molecule induced by anti-CD44 monoclonal antibodies. Immunobiology.

[38] Barrett T.B., Shu G., Clark E.A. (1991). CD40 signaling activates CD11a/CD18 (LFA-1)-mediated adhesion in B cells. J. Immunol..

[39] Kassambara A., Reme T., Jourdan M., Fest T., Hose D., Tarte K., Klein B. (2015). GenomicScape: An easy-to-use web tool for gene expression data analysis. Application to investigate the molecular events in the differentiation of B cells into plasma cells. PLoS Comput. Biol..

[40] Hatzoglou A., Roussel J., Bourgeade M.F., Rogier E., Madry C., Inoue J., Devergne O., Tsapis A. (2000). TNF receptor family member BCMA (B cell maturation) associates with TNF receptor-associated factor (TRAF) 1, TRAF2, and TRAF3 and activates NF-kappa B, elk-1, c-Jun N-terminal kinase, and p38 mitogen-activated protein kinase. J. Immunol..

[41] Thorley-Lawson D.A., Gross A. (2004). Persistence of the Epstein-Barr virus and the origins of associated lymphomas. N. Engl. J. Med..

[42] Katira A., Knox K.A., Finney M., Michell R.H., Wakelam M., Gordon J. (1993). Inhibition by glucocorticoid and staurosporine of IL-4-dependent CD23 production in B lymphocytes is reversed on engaging CD40. Clin. Exp. Immunol..

[43] Lim S.H., Vaughan A.T., Ashton-Key M., Williams E.L., Dixon S.V., Chan H.T., Beers S.A., French R.R., Cox K.L., Davies A.J. (2011). Fc gamma receptor IIb on target B cells promotes rituximab internalization and reduces clinical efficacy. Blood.

[44] Beum P.V., Kennedy A.D., Williams M.E., Lindorfer M.A., Taylor R.P. (2006). The shaving reaction: Rituximab/CD20 complexes are removed from mantle cell lymphoma and chronic lymphocytic leukemia cells by THP-1 monocytes. J. Immunol..

[45] Selvaraj A., Prywes R. (2004). Expression profiling of serum inducible genes identifies a subset of SRF target genes that are MKL dependent. BMC Mol. Biol..

[46] Wang D.Z., Li S., Hockemeyer D., Sutherland L., Wang Z., Schratt G., Richardson J.A., Nordheim A., Olson E.N. (2002). Potentiation of serum response factor activity by a family of myocardin-related transcription factors. Proc. Natl. Acad. Sci. USA.

[47] Smith E.C., Teixeira A.M., Chen R.C., Wang L., Gao Y., Hahn K.L., Krause D.S. (2013). Induction of megakaryocyte differentiation drives nuclear accumulation and transcriptional function of MKL1 via actin polymerization and RhoA activation. Blood.

[48] Bros M., Haas K., Moll L., Grabbe S. (2019). RhoA as a Key Regulator of Innate and Adaptive Immunity. Cells.

[49] Alon R., Ley K. (2008). Cells on the run: Shear-regulated integrin activation in leukocyte rolling and arrest on endothelial cells. Curr. Opin. Cell Biol..

[50] Sandbo N., Kregel S., Taurin S., Bhorade S., Dulin N.O. (2009). Critical role of serum response factor in pulmonary myofibroblast differentiation induced by TGF-beta. Am. J. Respir. Cell Mol. Biol..

[51] Miranda M.Z., Bialik J.F., Speight P., Dan Q., Yeung T., Szaszi K., Pedersen S.F., Kapus A. (2017). TGF-beta1 regulates the expression and transcriptional activity of TAZ protein via a Smad3-independent, myocardin-related transcription factor-mediated mechanism. J. Biol. Chem..

[52] Elberg G., Chen L., Elberg D., Chan M.D., Logan C.J., Turman M.A. (2008). MKL1 mediates TGF-beta1-induced alpha-smooth muscle actin expression in human renal epithelial cells. Am. J. Physiol. Renal Physiol..

[53] Small E.M. (2012). The actin-MRTF-SRF gene regulatory axis and myofibroblast differentiation. J. Cardiovasc. Transl. Res..

[54] Fleige A., Alberti S., Grobe L., Frischmann U., Geffers R., Muller W., Nordheim A., Schippers A. (2007). Serum response factor contributes selectively to lymphocyte development. J. Biol. Chem..

[55] Rehmann J.A., LeBien T.W. (1994). Transforming growth factor-beta regulates normal human pre-B cell differentiation. Int. Immunol..

[56] Kehrl J.H., Roberts A.B., Wakefield L.M., Jakowlew S., Sporn M.B., Fauci A.S. (1986). Transforming growth factor beta is an important immunomodulatory protein for human B lymphocytes. J. Immunol..

[57] Kehrl J.H., Thevenin C., Rieckmann P., Fauci A.S. (1991). Transforming growth factor-beta suppresses human B lymphocyte Ig production by inhibiting synthesis and the switch from the membrane form to the secreted form of Ig mRNA. J. Immunol..

[58] Thomas S.M., Hagel M., Turner C.E. (1999). Characterization of a focal adhesion protein, Hic-5, that shares extensive homology with paxillin. J. Cell Sci..

[59] Matsuya M., Sasaki H., Aoto H., Mitaka T., Nagura K., Ohba T., Ishino M., Takahashi S., Suzuki R., Sasaki T. (1998). Cell adhesion kinase beta forms a complex with a new member, Hic-5, of proteins localized at focal adhesions. J. Biol. Chem..

[60] Chodankar R., Wu D.Y., Schiller B.J., Yamamoto K.R., Stallcup M.R. (2014). Hic-5 is a transcription coregulator that acts before and/or after glucocorticoid receptor genome occupancy in a gene-selective manner. Proc. Natl. Acad. Sci. USA.

[61] Lutz-Nicoladoni C., Wolf D., Sopper S. (2015). Modulation of Immune Cell Functions by the E3 Ligase Cbl-b. Front. Oncol..

[62] Fan Y., Qu X., Ma Y., Liu Y., Hu X. (2016). Cbl-b promotes cell detachment via ubiquitination of focal adhesion kinase. Oncol. Lett..

[63] Ryan P.E., Kales S.C., Yadavalli R., Nau M.M., Zhang H., Lipkowitz S. (2012). Cbl-c ubiquitin ligase activity is increased via the interaction of its RING finger domain with a LIM domain of the paxillin homolog, Hic 5. PLoS ONE.

[64] Gruber T., Hinterleitner R., Hermann-Kleiter N., Meisel M., Kleiter I., Wang C.M., Viola A., Pfeifhofer-Obermair C., Baier G. (2013). Cbl-b mediates TGFbeta sensitivity by downregulating inhibitory SMAD7 in primary T cells. J. Mol. Cell Biol..

[65] Dogan I., Bertocci B., Vilmont V., Delbos F., Megret J., Storck S., Reynaud C.A., Weill J.C. (2009). Multiple layers of B cell memory with different effector functions. Nat. Immunol..

[66] Lutz J., Dittmann K., Bosl M.R., Winkler T.H., Wienands J., Engels N. (2015). Reactivation of IgG-switched memory B cells by BCR-intrinsic signal amplification promotes IgG antibody production. Nat. Commun..

[67] Reth M. (1992). Antigen Receptors on B Lymphocytes. Annu. Rev. Immunol..

[68] Martin S.W., Goodnow C.C. (2002). Burst-enhancing role of the IgG membrane tail as a molecular determinant of memory. Nat. Immunol..

[69] Horikawa K., Martin S.W., Pogue S.L., Silver K., Peng K., Takatsu K., Goodnow C.C. (2007). Enhancement and suppression of signaling by the conserved tail of IgG memory-type B cell antigen receptors. J. Exp. Med..

[70] Liu W., Meckel T., Tolar P., Sohn H.W., Pierce S.K. (2010). Intrinsic properties of immunoglobulin IgG1 isotype-switched B cell receptors promote microclustering and the initiation of signaling. Immunity.

[71] Wan Z., Chen X., Chen H., Ji Q., Chen Y., Wang J., Cao Y., Wang F., Lou J., Tang Z. (2015). The activation of IgM- or isotype-switched IgG- and IgE-BCR exhibits distinct mechanical force sensitivity and threshold. Elife.

[72] Wang J., Lin F., Wan Z., Sun X., Lu Y., Huang J., Wang F., Zeng Y., Chen Y.H., Shi Y. (2018). Profiling the origin, dynamics, and function of traction force in B cell activation. Sci. Signal..

[73] Casan J.M.L., Wong J., Northcott M.J., Opat S. (2018). Anti-CD20 monoclonal antibodies: Reviewing a revolution. Hum. Vaccin Immunother..

[74] Marshall M.J.E., Stopforth R.J., Cragg M.S. (2017). Therapeutic Antibodies: What Have We Learnt from Targeting CD20 and Where Are We Going?. Front. Immunol..

[75] Pierpont T.M., Limper C.B., Richards K.L. (2018). Past, Present, and Future of Rituximab-The World’s First Oncology Monoclonal Antibody Therapy. Front Oncol.

