# Supplementary material for: Pharmacogenomic Analyses Implicate B Cell Developmental Status and MKL1 as Determinants of Sensitivity toward Anti-CD20 Monoclonal Antibody Therapy
Source: Cells. 2023 Jun 7;12(12):1574. doi: 10.3390/cells12121574 (PMC10297299; doi:10.3390/cells12121574)
Supplement: Supplementary file 1 [file cells-12-01574-s001.zip › Supplementary Captions.pdf]

Supplementary Captions:

Figure S1: Scatterplots of viability data for rituximab, ofatumumab, and obinutuzumab;
Figure S2: Manhattan plots;
Figure S3: Characterization of MKL1 knockdowns;
Figure S4: Gene ontology (GO) analysis and gene set enrichment analysis (GSEA);
Figure S5: Increased homotypic aggregation in resistant cells;
Figure S6: Surface immunoglobulin isotype expression correlates with sensitivity;
Figure S7: Separation of a lymphoblastoid cell line based on Ig isotype results in subpopulations with different sensitivity to anti-CD20 mAbs;
Figure S8: Prednisolone pretreatment increases sensitivity toward anti-CD20 mAb;
Figure S9: CD20 surface expression following prednisolone and anti-CD20 mAb treatment.
Figure S10: Potential protective mechanisms: endocytosis; excision; and membrane repair
Table S1: List of primers used for qPCR;
Table S2: Summary of viability results;
Table S3: Gene expression levels that are significantly correlated with sensitivity;
Table S4: List of genes interrogated during supervised gene expression analysis;
Table S5: Supervised gene expression analysis for ofatumumab+complement;

Figure S1: Scatterplots of viability data for rituximab, ofatumumab, and obinutuzumab. Scatter plots comparing viabilities of cells treated with anti-CD20 antibodies, rituximab (rtx), ofatumumab (ofat), or obinutuzumab (obin) using 645 lymphoblastoid cell lines from The 1000 genomes project either in the presence of human serum as a complement source or under normal culture conditions without complement. Viability is expressed relative to cells without antibody added. Each data point represents the average of one cell line assayed twice as quadruplicates.

Figure S2: Manhattan plots of MAGWAS $-\log_{10}$ (p-value) over 22 autosomes for the association of genotype and cell viability. The blue and red lines indicate the thresholds for the genome-wide suggestive significance level of 10^{-6} and the genome-wide significance level of 10^{-8}, respectively.

Figure S3: Characterization of MKL1 knockdown cell lines. A) Western blotting of MKL1 knockdown cell lines. Cell lines were prepared using a pool of 5 individual *MKL1* shRNAs followed by puromycin selection on the shRNA-treated population of cells. MKL1 protein levels are shown by western blot for each pair of cell lines; control cells and their respective MKL1 knockdown (shMKL1). MKL1 can be seen as the green, and GAPDH as the red immunoreactive bands. The respective uncropped images are provided below the cropped images. Immunoreactive bands were quantitated using NIH ImageJ. MKL1 levels were normalized with a GAPDH loading control. Per cent (%) reduction is expressed as one minus the ratio of knockdown relative to control levels times 100%. B,D) Pairs of control (empty vector) cell lines (green bars) along with the corresponding shMKL1 cell lines (blue bars) are shown as an average of triplicates ± stdev. B) Cell growth rates were estimated using the Alamar Blue assay. Growth rates are presented as an average of absorbance divided by number of hours taken at 4 separate time points from 24- to 96h following cell seeding. C) The results from graph B are expressed as a ratio of shMKL1 / control then averaged (± stdev) for groups of cells. D) Viability assay following 72h treatment with mitomycin C (MitoC, 0.5uM); doxorubicin (Dox, 125 nM); vincristine (Vinc, 20nM); prednisolone (Pred, 1uM); arsenic

trioxide (Ars, 5uM), or 5-fluorouracil (5FU, 125 uM). Viability is expressed relative to cells without drug treatment (treatment / control). E) The viability results from figure D are expressed as a ratio of shMKL1 / control then averaged (± stdev) for each compound. Statistical significance symbols are ns: $p > 0.05$, *: $p \leq 0.05$, **: $p \leq 0.01$, ***: $p \leq 0.001$.

Figure S4: Gene ontology (GO) and gene set enrichment analysis (GSEA). Comparison of differentially expressed genes (DEGs) between sensitive (1st quantile) and resistant (4th quantile) LCLs toward ofatumumab+complement and represented within their biological process group. The number of genes involved in that process are indicated by circle size while the Gene Ratio is the percentage of total DEGs in the given GO term. The color gradient indicates the adjusted *p*-value significance level.

Figure S5: Increased homotypic aggregation in resistant cells. Cell aggregation is shown in a representative micrograph of a resistant cell line (GM18948) and a sensitive cell line (GM11828). The graph displays the average cell aggregation size (±stdev) for 6 resistant lines (blue bar) and 6 sensitive lines (green bar). $p \leq 0.001$.

Figure S6: Surface immunoglobulin isotype expression correlates with sensitivity. Analysis of cell surface expression of IgM versus IgG by flow cytometry in three sensitive, three resistant, and three cell lines showing mixed a mixed population of cells. Anti-IgM / anti-IgG double-fluorescence analysis of lymphoblastoid cell lines.

Figure S7: Separation of a lymphoblastoid cell line based on Ig isotype results in subpopulations with different sensitivity to anti-CD20 mAbs. The cell line, GM12876 was sorted by flow cytometry based on surface expression of IgG into two populations: IgG^- (IgM^+, green bars) and IgG^+ (IgG^+, blue bars). A) The two sub-populations were then assayed by qPCR for relative mRNA expression of *IgG* and *IgM* showing enrichment for IgG in the IgG^+ population and enrichment for IgM in the IgG^- population. Shown is the average of quadruplicates ±stdev. B) The two sub-populations were assayed for sensitivity toward anti-CD20 mAbs in the presence of complement in a viability assay. Results shown are the average of triplicates ±stdev. Statistical significance symbols are: **: $p \leq 0.01$, ***: $p \leq 0.001$.

Figure S8: Prednisolone pretreatment increases sensitivity toward anti-CD20 mAb.Cell lines were pretreated with (green bar) or without (blue bar) 1uM prednisolone for 10 days then assayed for viability following treatment with ofatumumab in the presence of complement. Viability is expressed relative to -ofatumumab samples. Results shown are the average of triplicates ±stdev.

Figure S9: CD20 surface expression following prednisolone and anti-CD20 mAb treatment. A) Time course of CD20 surface expression following treatment with prednisolone. Lymphoblastoid cells were treated with prednisolone for the indicated times then cells assayed by flow cytometry for surface expression of CD20 based on the mean fluorescence intensity (MFI). The average fold increase of CD20 is shown for 8 lymphoblastoid cell lines (±sem). B) Histograms for CD20 expression for a single LCL (GM12813) cell line initially expressing low levels of CD20 then treated with prednisolone from 0 to 10 days. C,D) Effect of anti-CD20 mAb treatment on CD20 surface expression in a representative cell line (GM19189) with or without prednisolone pretreatment for 10 days. C) Histograms for CD20 expression in cells not exposed to prednisolone; comparing control cells (green) against mAb-treated cells (blue, ofatumumab + complement, 30 minutes) and against cells previously mAb treated then allowed to recover for 14 days (red). D) Histograms for prednisolone-treated cells; comparing control cells (green) against mAb-treated cells (blue).

Figure S10: Potential protective mechanisms: endocytosis; excision; and membrane repair. **Endocytosis**: A) To examine internalization of CD20, LCLs were pre-labelled with PE-conjugated anti-CD20 antibody (binding to the rituximab epitope). Cells were then treated with ofatumumab (binding a different epitope) in the presence of 25% human serum to initiate complement fixation followed by analysis of CD20 by flow cytometry. Control (untreated) cells are shown in blue and compared against mAb-treated cells (red) and pre-labeled then treated cells (green). Histograms for CD20 of the cell lines, GM12876 and GM19189 are shown. B) CD20 levels, based on the mean fluorescence intensity (MFI) are shown from the average of two cell lines (GM12876, GM19189) from two independent determinations ± stdev. **Excision**: C) Controls establishing differential centrifugation protocol of membrane-bound CD20 released from cells. The 250xg fraction represents the cell pellet fraction. Centrifugation at 1500xg removes apoptotic debris. Cell-free CD20 is collected in the 15,000xg fraction. Any remaining CD20 is then precipitated with PEG-10000. Western blot for CD20 is shown (using an HRP-conjugated secondary antibody). Shown are fractions from the cell equivalent of $2x10^{-5}$ cells (-) untreated or (+) treated with 10ug/ml rituximab and 25% human serum for 30′. D) Release of CD20 from 6 sensitive and 6 resistant cell LCLs. Western blot of CD20 collected in the 15,000 x g fraction from LCLs treated (+) or untreated (-). The cell equivalent obtained from the 250 x g cell pellet is shown in the leftmost lane for the GM18640 cell line for reference. Bands were quantitated using NIH ImageJ and the averages for the two groups of cells are shown below ± stdev. E) The relative amounts of cell-associated CD20 is shown from a $2x10^{-5}$ cell pellet and compared against the amount of CD20 constitutively released by the same sample in overnight culture as well as the amount released in 30′ ± 10ug/ml rituximab with 25% human serum using 2 LCLs and 2 lymphoma lines. **Membrane repair**: F) Membrane integrity was evaluated using uptake of the cell-impermeant dye, ethidium homodimer-1 (EthD1) following treatment with mAbs in the presence of complement. Results are shown for rituximab (rtx, black trace), ofatumumab (ofat, blue trace), and obinutuzumab (obin, orange trace) over time. The fluorescence of untreated cells was subtracted as background from treated cells. Dye uptake is then presented as a percentage relative to a positive control using cells lysed with 0.1% saponin. G) The average EthD1 uptake at 10 min was compared for a group of 4 sensitive (GM01124, GM07006, GM11828, GM12828) and 4 resistant (GM00255, GM10839, GM11989, GM19114) LCLs for each of the anti-CD20 mAbs. Statistical significance symbols are ns: $p > 0.05$, *: $p \leq 0.05$, **: $p \leq 0.01$, ***: $p \leq 0.001$.

Table S1: List of primers used for qPCR

Table S2: Summary of viability results. A) Descriptive statistics summary of the viability data for the population of cells for each antibody: Rituximab (Rtx); Ofatumumab (Ofat); and Obinutuzumab (Obin). The correlation coefficient comparing viability data of pairs of antibodies is shown as R^2. B) The average viability for each cell line is shown. Response to antibody was assayed under normal media conditions (media) or in the presence of human serum as a complement source. Viability is expressed relative to samples without antibody. Each cell line was assayed twice as quadruplicates.

Table S3: Gene expression levels that are significantly correlated with sensitivity. The significant gene results ($p < 0.05$, corrected Bonferroni p-value) are shown for each respective antibody in bold. The p-value is also shown when $p > 0.05$ to 0.25 if p is < 0.05 for another antibody. Data is sorted based on drug then level of significance.

Table S4: Gene list used for supervised gene expression analysis.

Table S5 : Supervised gene expression analysis results for ofatumumab+complement. Results are shown where the corrected p-value is less than 0.05
