# Supplementary material for: Pharmacogenomic Analyses Implicate B Cell Developmental Status and MKL1 as Determinants of Sensitivity toward Anti-CD20 Monoclonal Antibody Therapy
Source: Cells. 2023 Jun 7;12(12):1574. doi: 10.3390/cells12121574 (PMC10297299; doi:10.3390/cells12121574)
Supplement: Supplementary file 1 [file cells-12-01574-s001.zip › Table S1-primer list.pdf]

**Table S1**: List of primers used for qPCR

| Gene/primer name | Sequence |
|---|---|
| CD20 for | GAGCCAATGAAAGGCCCTATT |
| CD20 rev | TGCTGCCAGGAGTGATCCGGAA |
| CD23 for | GGTATGCCTGTGACGACATGGA |
| CD23 rev | TTCAGGTCCAAGTTCCGAAGGC |
| CD40 for | CCTGTTTGCCATCCTCTTGGTG |
| CD40 rev | AGCAGTGTTGGAGCCAGGAAGA |
| CD69 for | GCTGGACTTCAGCCCAAAATGC |
| CD69 rev | AGTCCAACCCAGTGTTCCTCTC |
| GAPDH for | CTTTTGCGTCGCCAG |
| GAPDH rev | TTGATGGCAACAATATCCAC |
| J-region for (IgM/G) | MYGGTCACYGTCTCYTCAG |
| IGG rev | AAGACCGATGGGCCCTTG |
| IgM rev | GGGAATTCTCACAGGAGACG |
| IRF4 for | GAACGAGGAGAAGAGCATCTTCC |
| IRF4 rev | CGATGCCTTCTCGGAACTTTCC |
| MKL1 11-12 for | CAGAACAGCACCTCACTGACTG |
| MKL1 11-12 rev | CAGGCAGTGATCGCAACTTCAG |
| MKL1 13-14 for | CAGCAGCCCAAACAGCAGG |
| MKL1 13-14 var rev | GAAATTTCTGGGTTACCTCCG |
| MKL1 13-14 wt rev | CTGCTGAAATTTCTCCGCTCTG |
| PAX5 for | CTTGCTCATCAAGGTGTCAGGC |
| PAX5 rev | TGGCGACCTTTGGTTTGGATCC |
| TNFRSF17 for | TCTTTGGCAGTTTTCGTGCTAATG |
| TNFRSF17 rev | TTCTGCTCTCCTGAAGCCACCT |
| CD20 for | GAGCCAATGAAAGGCCCTATT |
| CD20 rev | TGCTGCCAGGAGTGATCCGGAA |
