# Supplementary material for: Pharmacogenomic Analyses Implicate B Cell Developmental Status and MKL1 as Determinants of Sensitivity toward Anti-CD20 Monoclonal Antibody Therapy
Source: Cells. 2023 Jun 7;12(12):1574. doi: 10.3390/cells12121574 (PMC10297299; doi:10.3390/cells12121574)
Supplement: Supplementary file 1 [file cells-12-01574-s001.zip › Table S2 summary of viability.pdf]

Table S2: Table summarizing viability results. A) Descriptive statistics summary of the viability data for the population of cells for each antibody: Rituximab (Rtx); Ofatumumab (Ofat); and Obinutuzumab (Obin). The correlation coefficient comparing viability data of pairs of antibodies is shown as R^2. B) The average viability for each individual cell line is shown. Response to antibody was assayed under normal media conditions (media) or in the presence of human serum as a complement source. Viability is expressed relative to samples without antibody. Each cell line was assayed twice as quadruplicates.

A)

Complement present	Antibody	Minimum viability	Maximum viability	Median viability	Rtx R^2	Ofat R^2	Obin R^2
+	Rituximab	11%	123%	64%	1.00	0.45	0.24
+	Ofatumumab	0%	129%	60%	0.45	1.00	0.44
+	Obinutuzumab	0%	75%	37%	0.24	0.44	1.00
-	Ofatumumab	41%	102%	62%	n/a	1.00	0.52
-	Obinutuzumab	38%	101%	64%	n/a	0.52	1.00

B)

Cell ID (GM:)	Obin_media	Obin_serum	Ofat_media	Ofat_serum	Ritux_serum
1112	0.682440704	0.432766615	0.737987758	0.805512622	0.854456466
1113	0.508259046	0.313378131	0.687532774	0.6212584	0.77275504
1124	0.5066357	0.203319502	0.582559168	0.090693539	0.276822762
1125	0.62332612	0.540250772	0.700473547	0.757223333	0.799563874
1133	0.640601314	0.429391504	0.528928752	0.619402985	0.690357676
1134	0.595328002	0.265313849	0.666000296	0.5158628	0.849776634
1136	0.634113274	0.44552152	0.611677236	0.474602559	0.686312524
1137	0.694130195	0.499042757	0.698510646	0.67368645	0.763880026
1139	0.551475127	0.387024609	0.513825454	0.63310962	0.68158091
1140	0.630931857	0.662763827	0.610924598	0.634570944	0.688183639
1148	0.642589482	0.284675615	0.606882888	0.465324385	0.533557047
1149	0.683605202	0.621212121	0.577989927	0.856506239	0.896613191
1250	0.606759928	0.499777558	0.600201776	0.68445037	0.739531043
1251	0.521298966	0.267019018	0.56070325	0.598429815	0.584120992
1253	0.555873629	0.289280745	0.535106527	0.514549655	0.752287324
1254	0.740443605	0.593521279	0.672681342	0.819192193	0.998915695
1256	0.652086395	0.584076515	0.774298677	0.672353625	0.831846969
1257	0.593919134	0.233698239	0.536731933	0.376963351	0.510709186
1259	0.494455764	0.264710456	0.469034704	0.268240653	0.62075601
1260	0.540846158	0.309059633	0.651773402	0.604357798	0.611238532
1271	0.563931824	0.297357815	0.584253381	0.657113002	0.683833408
1272	0.563669538	0.497165722	0.606568507	0.822607536	0.854951651
1274	0.646323492	0.354819633	0.565984044	0.57717327	0.909520993

1275	0.586570561	0.452101859	0.618007081	0.498585287	0.549919159
1277	0.639100577	0.559879032	0.637390977	0.808266129	0.839919355
1278	0.621119538	0.560028001	0.534301192	0.663633182	0.734686734
1334	0.540451161	0.421070772	0.651489269	0.634306066	0.637178309
1341	0.669454802	0.479778877	0.638751722	0.611143439	0.644748327
1342	0.732363197	0.359499431	0.641630901	0.489761092	0.606939704
1344	0.655651585	0.58928945	0.795305544	1.161495014	0.713269646
1345	0.621821492	0.396398058	0.650833381	0.721187535	0.730591923
1347	0.584672509	0.388541232	0.701975345	0.605803551	0.689194827
1348	0.686783424	0.598903043	0.726595992	0.773531493	0.751061571
1350	0.600608316	0.588027592	0.557506285	0.702628386	0.729735365
1351	0.595697207	0.205643233	0.571098011	0.378766141	0.390881556
1353	0.70318622	0.553039939	0.68274562	0.645637242	0.703044179
1354	0.64528426	0.612036797	0.620342894	0.601501055	1.094821678
1356	0.58251634	0.28440367	0.557880845	0.346788991	0.478899083
1357	0.553170116	0.252709641	0.706839643	0.479178551	0.663148888
1359	0.664848659	0.311640415	0.510222307	0.577871687	0.581559739
1360	0.557651715	0.359303391	0.534432718	0.721356554	0.760769936
1365	0.575164398	0.263880597	0.530458276	0.621492537	0.620895522
1374	0.532670388	0.213546566	0.547868099	0.632173095	0.6420508
1375	0.482003918	0.096364686	0.567343371	0.297749567	0.386035776
1377	0.707591293	0.497810219	0.611575823	0.727493917	0.709120198
1378	0.619251485	0.530598053	0.673871012	0.657510431	0.734700974
1383	0.569653646	0.372229141	0.653589765	0.506475716	0.574595268
1384	0.605268622	0.413215339	0.686997145	0.596224189	0.619115044
1389	0.567576345	0.580444402	0	0.975412223	0.935759102
1390	0.517997575	0.183186246	0.670992061	0.268265524	0.341491653
1437	0.638727372	0.612137203	0.641744377	0.942282322	0.884564644
1438	0.681968629	0.5301407	0.665592172	0.689557248	0.760810636
1440	0.537562356	0.446813848	0.690318418	0.596462619	0.550175615
1441	0.608422435	0.647263682	0.675733133	0.902155887	0.501658375
1455	0.571085367	0.373962559	0.553354786	0.586495156	0.623399352
1456	0.718315764	0.311642095	0.673993771	0.501270971	0.571428571
1461	0.641460198	0.545378292	0.621678498	0.637476933	0.748196611
1462	0.573813232	0.361742424	0.592401977	0.480955387	0.732954545
1464	0.582187392	0.576875769	0.488984179	0.534440344	0.709102091
1465	0.567526755	0.194618834	0.526557678	0.435874439	0.505829596
1488	0.571323025	0.30935186	0.551110877	0.74595484	0.833988482
1489	0.472977065	0.165705296	0.58542124	0.558468799	0.735186156
1491	0.540477038	0.538405928	0.663866145	0.826314838	0.699258992
1492	0.534401405	0.151186791	0.486338198	0.398864809	0.393188854
1494	0.572767066	0.217293021	0.555656043	0.550720543	0.688895168
1495	0.554472665	0.296244986	0.547282645	0.333311119	0.430154524

1497	0.551444375	0.357359326	0.529918272	0.630335114	0.729465856
1498	0.561573374	0.317199654	0.528517398	0.686257563	0.719101124
1550	0.597720207	0	0.502279793	0.663755459	0.84279476
1551	0.535683311	0.684536996	0.481497543	0.38147984	0.567567568
1789	0.569084882	0.355345912	0.530650778	0.707861635	0.745597484
1790	0.561050264	0.420985285	0.634694103	0.556621881	0.522499467
1791	0.636351266	0.481632653	0.635353352	0.702332362	0.619241983
6984	0.666491443	0.442384291	0.642382733	0.583420737	0.686374788
6985	0.594825421	0.33034212	0.641195679	0.558048233	0.752103197
6986	0.613542759	0.290472646	0.620193907	0.596017864	0.694268701
6994	0.656700784	0.421961479	0.649368528	0.760681868	0.800531326
7000	0.620839994	0.374147217	0.599670591	0.457091562	0.522202274
7037	0.626780071	0.455448065	0.763255433	0.79302444	1.087746096
7051	0.632342738	0.577405858	0.586031452	0.845711297	0.771443515
7056	0.45297456	0.392421415	0.57201344	0.5511698	0.542988374
7346	0.560271968	0.281797361	0.600075927	0.579970078	0.719620591
7347	0.645637645	0.381255184	0.673997471	0.539120818	0.600774122
7357	0.63968016	0.453788179	0.619297494	0.661642205	0.684252281
11829	0.546594427	0.350634987	0.61873065	0.126903553	0.362098139
11830	0.672746485	0.262464986	0.658825691	0.510084034	0.540896359
11831	0.601785799	0.378895815	0.673690178	0.623775601	0.623775601
11832	0.634955151	0.371483376	0.640554499	0.717391304	0.704603581
11840	0.565137615	0	0.529408767	0.344827586	0.501668521
11881	0.514193835	0.288899901	0.52376728	0.49900892	0.573835481
11892	0.660479244	0.68149398	0.608119184	0.724058593	0.750287599
11893	0.661506056	0.362049336	0.565806565	0.681973435	0.55597723
11894	0.537701549	0.195370046	0.534460955	0.728165556	0.762188706
11918	0.554136373	0.288507109	0.637649833	0.317831754	0.561018957
11919	0.577806195	0.192099567	0.525234442	0.557088745	0.605519481
11920	0.929830069	0.52528312	0.878080163	0.832285653	0.72458209
11930	0.507307963	0.405761317	0.530213492	0.602057613	0.613580247
11931	0.603675059	0.496845006	0.558557694	0.709061901	0.851973726
11992	0.628815667	0.343364365	0.548119366	0.40073609	0.627192033
11993	0.679146526	0.470358367	0.620008372	0.885973462	0.75472659
11994	0.549640547	0.313043478	0.579037306	0.750197628	0.586824769
11995	0.867339379	0.443858011	0.971397037	0.588028056	0.723741199
12003	0.635004971	0.386728875	0.616331096	0.537325039	0.597459824
12004	0.555210072	0.422356717	0.555508587	0.614463671	0.672877049
12005	0.604923703	0.586459286	0.605534079	0.904849039	0.936413541
12006	0	0.583354009	0	0.7747786	0.79076217
12043	0.67252945	0.370634921	0.684827661	0.495238095	0.532936508
12044	0.639621881	0.427951389	0.678374625	0.596354167	0.542100694
12144	0.710369278	0.420572551	0.663963774	0.614931238	0.607914679

12154	0.709329454	0.684980674	0.780281613	0.909718388	0.793944414
12155	0.591212158	0.551762606	0.562650168	0.904804403	0.75292042
12156	0.624846484	0.512613522	0.640581824	0.592330979	0.572149344
12234	0.675462573	0.223313565	0.607570384	0.686644308	0.725185382
12249	0.708207854	0.465527066	0.655181577	0.634567901	0.667426401
12272	0.657504733	0.414634146	0.653456943	0.379512195	0.461463415
12273	0.646994012	0.525686553	0.637700599	0.641690341	0.700284091
12275	0.629861147	0.196321321	0.569810934	0.521771772	0.68714858
12286	0.746491255	0.468853545	0.648428264	0.912193506	0.91119947
12287	0.884482247	0.503340132	0.921995264	0.769708968	0.76802309
12341	0.611313474	0.25876659	0.633818431	0.46443561	0.447253308
12347	0.578792596	0.380566802	0.481635242	0.791093117	0.732793522
12348	0.657034259	0.538327335	0.671316021	0.717233868	0.730300076
12399	0.638739188	0.094478528	0.596666818	0.612269939	0.628220859
12400	0.627137454	0.367873347	0.587344643	0.421333201	0.594716512
12413	0.679570387	0.60691584	0.706564815	0.649885584	0.695652174
12414	0.669346098	0.370293966	0.623825867	0.705002579	0.824651882
12489	0.694098872	0.384379001	0.64502557	0.46453265	0.386939821
12546	0.608991902	0.375585522	0.708209997	0.512562101	0.587508872
12717	0.59178903	0.4106814	0.590208731	0.030156538	0.313996317
12748	0.713186507	0.568732048	0.724003345	0.64792778	0.610997128
12749	0.68014495	0.609591393	0.780775849	0.891724848	0.838502919
12750	0.583410066	0.434876502	0.662650285	0.671885545	0.695370495
12751	0.615986779	0.273547094	0.563451335	0.438376754	0.491482966
12760	0.656699506	0.282506176	0.668408297	0.651021783	0.526835841
12761	0.608076542	0.512807109	0.63655563	0.589475518	0.712493466
12762	0.547903254	0.390769231	0.570871759	0.486769231	0.470769231
12763	0.658040949	0.325814219	0.767013537	0.480420732	0.495374477
12775	0.618139293	0.412260967	0.588773389	0.661417323	0.623734533
12776	0.615549666	0.177820268	0.586015398	0.407903123	0.407265774
12777	0.8795926	0.528389569	0.976537191	0.910814265	0.952278628
12778	0.651446759	0.184538767	0.595138889	0.385571344	0.390597158
12812	0.625719856	0.378310802	0.713464919	0.643611018	0.52093244
12813	0.496891853	0.333205521	0.639784282	0.432898773	0.358703988
12814	0.690278855	0.417380137	0.667047728	0.566780822	0.676369863
12815	0.641543376	0.317394317	0.614590118	0.58038808	0.428620929
12827	0.829679017	0.463932521	0.806342791	0.302761906	0.533198153
12828	0.586903545	0.308580858	0.55927207	0.484323432	0.577557756
12829	0.664558674	0.547669814	0.634528238	0.589399939	0.643009443
12830	0.423099881	0.383156114	0.473338639	0.460729821	0.758699855
12842	0.725527729	0.412332838	0.54543521	0.773402675	0.686478455
12843	0.606234517	0.332508834	0.5835673	0.444169611	0.502473498
12872	0.614287096	0.298835705	0.694331592	0.54894351	0.416990082

12873	0.670705188	0.447382337	0.582133858	0.634902168	0.634584876
12874	0.595682419	0.361043983	0.54562723	0.579748671	0.355969067
12889	0.555815109	0.113366337	0.567445328	0.245049505	0.431683168
12890	0.655933973	0.300098912	0.6734847	0.361028684	0.52007913
12891	0.711717122	0.418122977	0.651806846	0.557065804	0.683063646
12892	0.683015403	0.476219699	0.634094419	0.54740718	0.677201596
18486	0.574617647	0.313164827	0.645343259	0.623888565	0.586843049
18487	0.492044064	0.123076923	0.530116601	0.40361991	0.502262443
18488	0.690170739	0.346759259	0.61338553	0.579166667	0.765277778
18489	0.539997597	0.336962514	0.602691337	0.457965821	0.499448732
18498	0.517188616	0.341902735	0.628413071	0.546913378	0.644178811
18499	0.632235669	0.58446029	0.568439816	0.731989181	0.658716499
18501	0.619054836	0.378108031	0.654719507	0.471420406	0.482280652
18502	0.674159579	0.534632854	0.698832186	0.641180924	0.746396937
18504	0.672495127	0.716698656	0.670461286	0.661228407	0.886628279
18505	0.677509377	0.38878327	0.639129782	0.496514575	0.741339248
18507	0.558427762	0.026348808	0.594458215	0.257214555	0.319113342
18508	0.528221027	0.20856202	0.539071761	0.486278814	0.566410538
18516	0.693026328	0.506053269	0.685068101	0.258419547	0.491965661
18517	0.563957337	0.334513274	0.627657227	0.500884956	0.497935103
18519	0.751943708	0.376837652	0.753138166	0.593442623	0.742464305
18520	0.473634698	0.506423259	0.528197257	0.574036511	0.620689655
18522	0.614868288	0.415	0.557814083	0.618	0.972
18523	0.648158671	0.430952036	0.660009532	0.754166063	0.841109984
18525	0.646637325	0.538781959	0.714105523	0.791151968	0.754811836
18526	0.674492124	0.446259673	0.71311543	0.916881628	0.928537308
18527	0.658194324	0.401249024	0.654715788	0.523289097	0.786104606
18528	0.577968944	0.289727127	0.639279726	0.49317817	0.585473515
18530	0.618198138	0.361022364	0.585212611	0.865814696	0.881789137
18531	0.646865443	0.026625502	0.610626911	0	0.473922902
18532	0.555700709	0.31672917	0.670727326	0.401108756	0.599461927
18533	0.689920935	0.464163823	0.589295488	0.755972696	0.756825939
18534	0.574255157	0.142857143	0.631168831	0.443064182	0.380952381
18535	0.617595692	0.202735318	0.608426721	0.279163315	0.390185036
18536	0.712912237	0.567263221	0.710735406	0.54936658	0.706414639
18537	0.646930065	0.335471456	0.56807486	0.66132136	0.584348942
18538	0.560062565	0.493513514	0.581717066	0.547387387	0.523963964
18539	0.548146733	0.387938794	0.615240097	0.588883888	0.661116112
18541	0.555988155	0.398470688	0.615557611	0.760407816	0.63126593
18542	0.635297144	0	0.628093645	0.110497238	0.406629834
18543	0.623756742	0.453104199	0.689024692	0.700714026	0.636330631
18544	0.550072104	0.169770774	0.62137478	0.767191977	0.648997135
18545	0.609470113	0.531831962	0.697136014	0.788220009	0.699870074

18546	0.644687898	0.317073171	0.61110828	0.630589431	0.576727642
18547	1.008270589	0.329801807	0.949290661	0.699019818	0.701367786
18548	0.648526268	0.390289256	0.622792215	0.639256198	0.588429752
18549	0.539295531	0	0.588465845	0.531677019	0.501863354
18550	0.576946681	0.25	0.63773871	0.564710611	0.508440514
18552	0	0.486726457	0.539270881	0.652313526	0.727688908
18553	0.673428174	0.351378446	0.757438406	0.626566416	0.613784461
18555	0.560206268	0.200956938	0.589271364	0.299043062	0.41507177
18557	0.613038546	0.422071636	0.594438435	0.551306873	0.617618587
18558	0.656439705	0.291830494	0.586054143	0.54405126	0.647444299
18559	0.649734982	0.321782178	0.608215548	0.624752475	0.614851485
18560	0.71691096	0.457413249	0.638864581	0.758675079	0.784700315
18561	0.53826619	0.048021895	0.634883989	0.381628386	0.292343387
18562	0.60003775	0.23039923	0.574745187	0.60990861	0.667147667
18563	0.602966725	0.283231084	0.504699541	0.579243354	0.446148603
18564	0.630994627	0.333641405	0.633673949	0.384781269	0.471041282
18565	0.57205298	0.340815563	0.607593819	0.69509914	0.64983165
18566	0.74789328	0.600914962	0.742326062	0.773950484	0.79426803
18567	0.554544068	0.295217716	0.522249911	0.798226164	0.257206208
18570	0.609908313	0.320363165	0.590415286	0.660181582	0.715953307
18571	0.721371691	0.434935897	0.624747645	0.791025641	0.895726496
18572	0.638902526	0.475521499	0.623138603	0.5700298	0.62962963
18573	0.581063971	0.482401461	0.609168504	0.715262733	0.614220685
18574	0.693420188	0.296690307	0.624873005	0.552009456	0.655437352
18576	0.665038252	0.279818074	0.606415338	0.5017215	0.450714104
18577	0.641511387	0.583469722	0.641563147	0.51309329	0.495090016
18579	0.626181069	0.263417643	0.731527611	0.503392967	0.599629858
18582	0.51300212	0.257236246	0.643333506	0.209020902	0.349834983
18592	0.624462398	0	0.562201053	0.171253823	0.675992684
18593	0.697357324	0.367080745	0.696092213	0.38757764	0.571428571
18595	0.588458298	0.270880361	0.564207486	0.673814898	0.700902935
18596	0.628896106	0.210737179	0.599109813	0.666666667	0.955929487
18597	0.645822596	0	0.703086526	0.30698152	1.079055441
18599	0.520762215	0.187554019	0.551892801	0.61624892	0.643906655
18602	0.590744899	0.344917463	0.63863126	0.598609904	0.692730959
18603	0.652360515	0.427268363	0.656843109	0.664426308	0.62506001
18605	0.588409198	0.275390625	0.532144533	0.493164063	0.63671875
18606	0.580618062	0.1842871	0.623074483	0.55771096	0.646944714
18608	0.565194482	0.342533937	0.664303506	0.509049774	0.573755656
18609	0.690744391	0.268450933	0.640664602	0.53163017	0.508515815
18610	0.572792223	0.24512567	0.563422347	0.491056771	0.462522832
18611	0.672262933	0.344282238	0.683254949	0.561638281	0.484184915
18612	0.647403378	0.099665552	0.590868678	0.309698997	0.438795987

18613	0.684375178	0.08520764	0.642739321	0.421349661	0.64549034
18614	0.531127146	0.249763404	0.578800697	0.629394813	0.399423631
18615	0.635674583	0	0.552371328	0.227817746	0.278177458
18616	0.597322809	0.175984122	0.598665473	0.539530268	0.517697651
18617	0.598225444	0.035266458	0.56464009	0.684952978	0.956896552
18618	0.570965251	0	0.543706564	0.42172524	0.576996805
18619	0.673765494	0.006769826	0.744041658	0.234042553	0.473887814
18620	0.64418409	0.557632399	0.609618746	0.59709242	0.332294912
18621	0.590515708	0.088285229	0.558743331	0.482173175	0.148556876
18622	0.68735553	0.350508838	0.601448384	0.533690412	0.664167113
18623	0.708614122	0.214023943	0.660445356	0.429025165	0.52821891
18624	0.631701991	0.157078987	0.706769428	0.729061103	0.64023845
18625	0.601731738	0.278707572	0.623014507	0.459194497	0.592647707
18626	0.683857486	0.457091925	0.69466803	0.774934043	0.927442802
18627	0.588211195	0	0.560454755	0.201492537	0.468113976
18628	0.604721129	0.170661157	0.628280491	0.688842975	0.716528926
18630	0.709120381	0.333241531	0.690628205	0.561369687	0.610208391
18631	0.611730428	0.217464316	0.671712693	0.451721243	0.661628883
18632	0.611824459	0.260670295	0.628887748	0.66571183	0.794614724
18633	0.597802198	0.113967438	0.555156382	0.405312768	0.680377035
18634	0.577870216	0	0.596838602	0.430978613	0.772521063
18635	0.550457347	0.205761317	0.552893219	0.59304984	0.595793324
18636	0.595099236	0.259932585	0.561232335	0.619380152	0.721873234
18637	0.619495192	0.412022139	0.833058417	0.665722744	0.73677436
18638	0.744910563	0.448985407	0.652424075	0.997577958	0.932182819
18639	0.691111911	0.318066158	0.581935948	0.704834606	0.347328244
18640	0.622446889	0.061283492	0.659617906	0	0.208804945
18641	0.593318848	0.08749581	0.541390549	0.586322494	0.75527992
18642	0.618254555	0	0.649780406	0.310030395	0.594224924
18643	0.538200414	0	0.543049861	0.741452991	0.492521368
18645	0.570690149	0.079967414	0.534009666	0.553487121	0.562489396
18647	0.598128961	0.178793419	0.665996043	0.469835466	0.586471664
18740	0.614605279	0.212555442	0.642582724	0.578300921	0.681337427
18745	0.675480693	0.314143921	0.684391394	0.693796526	0.65707196
18747	0.609447109	0.012839879	0.593566193	0.498334372	0.120090634
18748	0.684061753	0.132701422	0.585282515	0.705730289	0.737613098
18749	0.602913418	0.088951311	0.619696348	0.502808989	0.378277154
18853	0.586898085	0.077257364	0.556798195	0.563495896	0.450024143
18856	0.534293692	0	0.593169587	0.173441734	0.353206865
18858	0.545876335	0	0.601879666	0.037721893	0.586538462
18861	0.816549534	0.387343778	0.829103074	0.739063898	0.744681273
18867	0.506348342	0.295854376	0.571637388	0.427377911	0.534818882
18868	0.673888012	0.34272441	0.676274144	0.299413607	0.425607363

18870	0.742554281	0.438593987	0.733876847	0.748126092	0.686531681
18873	0.642323705	0.319562791	0.709159752	0.560050352	0.674582102
18910	0.616086032	0.328518468	0.679786822	0.507483374	0.635503819
18912	0.617489492	0.109476388	0.647770147	0.197640663	0.445179928
18916	0.611297345	0.19666065	0.551928968	0.42045732	0.492187146
18917	0.681798172	0.372274283	0.654724667	0.724838102	0.782045475
18923	0.570682997	0.053047699	0.653465834	0.507681741	0.68057315
18924	0.627969332	0.252197481	0.687646763	0.578855283	0.63364842
18933	0	0.335644421	0	0.428923986	0.533717144
18934	0.653088414	0	0.55674448	0.389956492	0.121202568
18939	0.668164864	0.266473982	0.640066437	0.294386965	0.845598681
18940	0.702129453	0.411235307	0.739717312	0.794093882	0.632259793
18941	0.667537665	0.537512015	0.722818433	0.653811061	0.80193194
18942	0.648218795	0.309184512	0.653732161	0.897811382	0.959874778
18943	0.563285988	0.224678203	0.594000209	0.628071293	0.836041013
18944	0.674824116	0.287806449	0.681137684	0.450228542	0.421624118
18945	0.588678092	0.06340786	0.564427111	0.427017866	0.459301946
18946	0.630231561	0.255674147	0.675930624	0.386123139	0.644537516
18947	0.536023796	0.270618557	0.675896005	0.67583047	0.838487973
18948	0.797834844	0.600174978	0.790465074	1.020086031	0.869313211
18949	0.590124005	0.25344332	0.709709883	0.551748649	0.617950237
18950	0.841103274	0.524218034	0.856786293	0.817108882	0.789088297
18951	0.683915879	0.226306038	0.636161337	0.618933963	0.588750443
18952	0.576027203	0.167771883	0.677415698	0.472811671	0.702917772
18953	0.72257129	0.381167837	0.836376795	0.651690804	0.67745885
18954	0.562931918	0	0.593991539	0.263925475	0.491364233
18955	0.585974629	0.257074819	0.645108015	0.545343862	0.667244471
18956	0.675880901	0.23414785	0.730305977	0.436127436	0.447571915
18957	0.550899782	0	0.532573267	0.367857935	0.547293911
18959	0.606742265	0.23083779	0.617650684	0.443850267	0.598930481
18960	0.720951852	0.418904591	0.652353032	0.611844593	0.598661362
18961	0.674521062	0.37685503	0.685839467	0.748049047	0.725338587
18962	0.495063134	0.176974162	0.603514999	0.752340749	0.744710807
18963	0.636602734	0.105363417	0.625078913	0.382765333	0.500751365
18964	0.578010582	0.110448103	0.538274466	0.45031576	0.580212476
18965	0.508664245	0	0.507495094	0.458225221	0.738453555
18966	0.676275871	0.348002229	0.724654109	0.517430789	0.550400153
18967	0.595462019	0.487708168	0.618612299	0.6740682	0.901665345
18968	0.607547969	0.07321536	0.661105978	0.374869283	0.586441293
18969	0.639398906	0.487726186	0.665782199	0.691844946	0.776271979
18970	0.716923357	0.484175605	0.726205058	0.846446567	0.753169928
18971	0.893751992	0.592855623	0.901672427	0.714500392	0.722144735
18972	0.619205683	0.276939665	0.707906908	0.480963177	0.614789074

18973	0.5580651	0.060267299	0.596974141	0.430899722	0.548275929
18974	0.628596944	0.194344525	0.629327243	0.393616861	0.710911163
18975	0.698024509	0.016624685	0.690312698	0.465491184	0.436775819
18976	0.634646958	0.313361297	0.681110692	0.632009279	0.651638007
18977	0.606751112	0.374797041	0.596809242	0.625535159	0.636610489
18978	0.676474119	0.396829041	0.747748701	0.601234238	0.69516903
18979	0.692699888	0.352551599	0.64766816	0.601317748	0.880648703
18980	0.582109072	0.174461972	0.655665584	0.226681842	0.428717028
18981	0.641524812	0.420713914	0.688258423	0.563435878	0.730265913
18982	0.678758957	0.230460053	0.660016718	0.628387293	0.620599059
18983	0.588676871	0.198825292	0.603396058	0.486485467	0.575787717
18984	0.598783362	0.359506901	0.632721951	0.725920747	0.744813105
18985	0.546679645	0.320984466	0.620775154	0.720221465	0.560528666
18986	0.631462455	0.447050605	0.707190323	0.542642016	0.600159882
18988	0.671865042	0.191577494	0.676163699	0.653089403	0.636865723
18989	0.697967022	0.268225479	0.66867304	0.423932629	0.506465313
18990	0.67203607	0.353803295	0.635434068	0.704102456	0.764262357
18991	0.656553668	0.374566018	0.634602422	0.635639334	0.727455638
18993	0.625449843	0.101068999	0.621907332	0.445092323	0.449951409
18994	0.659826809	0.626680551	0.634762057	0.713323181	0.753207683
18995	0.747175773	0.53066386	0.704792711	0.766298858	0.841867283
18997	0.548803454	0.227319062	0.600431767	0.682976555	0.822629969
18998	0.62369168	0.554162936	0.584663828	0.730975828	0.615487914
18999	0.654217259	0.466666667	0.661643539	0.607843137	0.567755991
19000	0.565163576	0.30835041	0.632324371	0.354711346	0.444335059
19001	0.599113806	0.166051661	0.565598348	0.733790195	0.762256194
19002	0.678531844	0.563369886	0.706581976	0.832560101	0.700021088
19003	0.656964147	0.447318268	0.711050243	0.657228424	0.728832017
19004	0.677976146	0.178133836	0.580094229	0.512723845	0.75871819
19005	0.642239977	0.290780142	0.626152344	0.602836879	0.693169093
19007	0.687080943	0.479077711	0.647404438	0.70509536	0.72018218
19009	0.611186275	0.354403409	0.627460784	0.684422348	0.608664773
19010	0.668114819	0.472071289	0.629660975	0.774614214	0.885459683
19012	0.67141326	0.271707029	0.620349104	0.527466037	0.654459539
19027	0.608558292	0.381997313	0.663761248	0.676892073	0.714957456
19028	0.982886293	0.538085457	0.993199113	0.791103505	0.853745218
19031	0.508963021	0.270123861	0.57157341	0.578293786	0.604078584
19035	0.602542656	0.109361545	0.607895617	0.06684492	0.411764706
19036	0.601281483	0.05351932	0.605645375	0.201296973	0.806481645
19038	0.605282831	0.469667319	0.644717169	0.432485323	0.692759295
19041	0.675475874	0.146010187	0.61218763	0.585314092	0.412988115
19044	0.758688559	0.535860368	0.700965003	0.754014728	0.759984829
19046	0.503039514	0.40606339	0.531525212	0.49701424	0.602204869

19054	0.739855426	0.620473096	0.662920913	0.882231291	0.876677136
19055	0.684323843	0.334553776	0.71511565	0.572997712	0.632951945
19056	0.636629842	0.112033195	0.629841608	0.283817427	0.310373444
19057	0.647564578	0.350495985	0.650746156	0.598488427	0.546055739
19058	0.589965195	0.349889625	0.613390169	0.718175129	0.653789551
19059	0.725467864	0.520423601	0.656252427	0.835098336	0.775424441
19060	0.916360926	0.384639112	0.997735611	0.700801974	0.694941394
19062	0.600853595	0.445777573	0.699612002	0.69589294	0.712505768
19063	0.625380093	0.291452991	0.615987567	0.466666667	0.62991453
19064	0.627404415	0.351864884	0.606162558	0.804363125	0.825123153
19065	0.458467618	0	0.540122685	0.544011544	0.576479076
19066	0.631232782	0.27990092	0.592045455	0.640481246	0.689667374
19067	0.555756091	0.323357413	0.620256972	0.629858961	0.7000344
19068	0.649804661	0.461305925	0.658258117	0.708383716	0.981794975
19070	0.6829806	0.535578947	0.667989418	0.941894737	0.749473684
19072	0.713671704	0.461928105	0.708032194	0.827941176	0.709150327
19074	0.624003432	0.248898991	0.623967681	0.568688734	0.445091632
19075	0.686133287	0.450634205	0.660436483	0.696485605	0.494113294
19076	0.605251811	0.24	0.567911349	0.446984127	0.86984127
19077	0.543465257	0	0.558166042	0.149343131	0.36533788
19078	0.622597734	0.152955767	0.580720003	0.470442332	0.479950393
19079	0.734991981	0.593592301	0.755407221	0.717873489	0.779838047
19080	0.667058983	0.474872346	0.669186547	0.790110185	0.96371943
19081	0.711367108	0.384180791	0.692718941	0.588512241	0.746704331
19082	0.791457587	0.610324791	0.765591398	0.881182956	0.771901073
19083	0.716344861	0.438123167	0.748776421	0.648680352	0.835777126
19084	0.661145336	0.334277264	0.607114606	0.785350198	0.623749292
19085	0.710009481	0.393172318	0.60263443	0.732234092	0.573385973
19086	0.63113791	0.538178672	0.674551639	0.692623837	0.661258923
19087	0.539719148	0.263067695	0.52699248	0.605826907	0.718937446
19088	0.573797621	0.181571816	0.682847785	0.427168022	0.513550136
19092	0.715395823	0.552040348	0.702646916	0.640531866	0.770747364
19093	0.622433634	0.321726336	0.582679482	0.718979892	0.584600294
19095	0.538037798	0.23908005	0.585414438	0.5749688	0.601711535
19096	0.587169111	0.36324544	0.65986624	0.709904546	0.512191664
19098	0.66758175	0.476339654	0.635974928	0.671723001	0.711788953
19099	0.71387511	0.519026344	0.742362431	1.003235249	0.786781698
19107	0.661716172	0.61056437	0.660571159	0.838923479	0.510343442
19108	0.623250453	0	0.498348713	0.269396254	0.301537874
19113	0.673989321	0.370827286	0.649478769	0.332365747	0.480406386
19114	0.958584471	0.603014191	0.900952935	0.942133532	0.963314843
19116	0.542366572	0.233707865	0.58940952	0.514606742	0.573033708
19117	0.660313009	0.172698244	0.577930198	0.445449707	0.638903672

19118	0.690497305	0.483148295	0.749641858	0.677835052	0.73037272
19119	0.709954594	0.510661921	0.669586774	0.63166768	0.794662261
19121	0.67481203	0.194394674	0.563771167	0.396475786	0.568061476
19130	0.712264421	0.519974716	0.785673369	0.794184576	0.608217446
19131	0.661288218	0.28526971	0.639368503	0.438278008	0.519190871
19137	0.675550232	0.393530997	0.659704547	0.753680282	0.685050798
19138	0.6593997	0.559030348	0.704293021	0.828830496	0.840118431
19141	0.600911298	0.330845434	0.720662465	0.531686796	0.596824535
19143	0.633593553	0.338189845	0.573491144	0.66799117	0.694922737
19144	0.652285438	0.459989675	0.659602203	0.556014455	0.745482705
19146	0.522902664	0.341063433	0.620702819	0.597315456	0.575424507
19147	0.596859239	0.361531612	0.615657955	0.546156129	0.757494806
19149	0.633615478	0.48544314	0.623941959	0.814701625	0.746501528
19150	0.606883597	0.318165532	0.641906521	0.566463633	0.677893228
19152	0.553398589	0.49089864	0.566141701	0.785016287	0.854378233
19153	0.746163046	0.253636364	0.59253538	0.459090909	0.570454545
19159	0.83453591	0.751234389	0.74649838	0.728289283	0.807435376
19160	0.553110344	0.508416352	0.683347936	0.616283064	0.643421505
19171	0.605286912	0.350599315	0.69245648	0.425941781	0.480736301
19172	0.525396447	0.134927066	0.554608322	0.315640194	0.482576985
19184	0.583567779	0.462022661	0.611816042	0.328787243	0.583088544
19185	0.617374172	0.440695836	0.736987283	0.537427517	0.581444386
19189	0.528569806	0.074688797	0.555927903	0.344628861	0.383817427
19190	0.540724591	0.486054972	0.619401378	0.856911884	0.692805174
19197	0.6189795	0.57198083	0.696822927	0.773497668	0.78326519
19198	0.614270767	0.184266103	0.589394809	0.587117212	0.683210137
19200	0.696136985	0.323749416	0.711494895	0.332398317	0.501402525
19201	0.50623104	0.195562436	0.515618595	0.237358101	0.565531476
19204	0.593899646	0.34797136	0.5292005	0.489260143	0.802863962
19206	0.6073903	0.442405708	0.624977794	0.569826707	0.515800204
19207	0.604585352	0.227670753	0.549282939	0.347343841	0.609457093
19209	0.570668949	0.294824258	0.618117472	0.5678856	0.571670691
19210	0.540374633	0.398589065	0.608395396	0.610582011	0.840917108
19213	0.59644922	0.384814495	0.680663435	0.339085418	0.355478861
19214	0.618710958	0	0.609558859	0.32576229	0.698195395
19222	0.682945355	0.312317572	0.748894151	0.429071804	0.539988325
19223	0.579559023	0.160371877	0.534147895	0.377396862	0.563916328
19225	0.623801007	0.122806823	0.559614436	0.783957146	0.753178438
19235	0.661190465	0.205719163	0.596451031	0.416559966	0.714041827
19236	0.63247084	0.277484398	0.547685562	0.445991359	0.620739318
19247	0.637174601	0	0.68072338	0.047957371	0.204262877
19248	0.569089444	0.227544215	0.547835986	0.459387791	0.559283525
19256	0.611444805	0.28319209	0.607021104	0.60240113	0.738700565

19257	0.597796474	0.268163804	0.614756944	0.752972259	0.739321885
19307	0.76696924	0.179916318	0.623865349	0.521751933	0.531903766
19308	0.538228411	0.358346928	0.627881996	0.504078303	0.723219141
19309	0.720556488	0.532830739	0.606044615	0.677285992	0.624270428
19310	0.671330787	0.297800338	0.671633325	0.728003384	0.735194585
19311	0.603479702	0.321875	0.641259321	0.644034091	0.595170455
19312	0.617341641	0.321348315	0.634735202	0.675955056	0.534831461
19313	0.506403235	0.559346909	0.659617491	0.411117226	0.571271326
19314	0.661087455	0.154826066	0.667736652	0.687898089	0.6722195
19315	0.622160956	0.347211419	0.584167529	0.567031987	0.576060332
19316	0.618980593	0.223540146	0.636169759	0.318430657	0.665145985
19317	0.462668131	0.220111732	0.582349712	0.198882682	0.238175047
19318	0.601480174	0.207698816	0.636622065	0.182318105	0.475888325
19319	0.579955433	0	0.556876061	0.079088472	0.507372654
19321	0.671545148	0.35209826	0.645999645	0.473387922	0.502047083
19324	0.556730515	0.081300813	0.683084916	0.048780488	0.432520325
19327	0.498527312	0.344858689	0.586977865	0.124774504	0.397775105
19328	0.591544848	0.435440303	0.723154665	0.686115613	0.80713128
19334	0.707016057	0	0.603089759	0	0.10554951
19338	0.580233126	0.539060912	0.642911984	0.751880209	0.673622874
19350	0.684746834	0.623006283	0.697859343	0.807636539	0.865635573
19351	0.552245011	0.322531258	0.563913525	0.511610105	0.648890023
19352	0.829053907	0.453301447	0.85731135	0.615718814	0.750598965
19355	0.669220999	0.278	0.749322608	0.5676	0.6812
19359	0.605194317	0.450685139	0.606676215	0.641093426	0.663763231
19360	0.703034525	0.307534334	0.652488136	0.688486715	0.722757027
19371	0.678589336	0.307732772	0.612067525	0.634928985	0.731720147
19372	0.688321512	0.525512518	0.673073332	0.701742536	0.818143024
19374	0.503417246	0.238615665	0.605649846	0.310564663	0.345173042
19375	0.511740464	0.036180905	0.48902626	0.057286432	0.583919598
19376	0.593720038	0.298099762	0.63590604	0.45783848	0.484263658
19377	0.563672679	0.07591623	0.594298511	0.310209424	0.755235602
19379	0.670017263	0.470708574	0.689953778	0.81532453	0.94123117
19380	0.585515702	0.139772046	0.684609593	0.26034793	0.438512298
19381	0.657382713	0.408464567	0.625257607	0.641732283	0.863681102
19383	0.56069638	0.3952	0.59369942	0.3424	0.4248
19384	0.625721396	0.354934982	0.617938264	1.000213174	0.578767853
19385	0.602317246	0.219529579	0.60898558	0	0.516393443
19390	0.616125448	0.428838077	0.709593777	0.625291647	0.760615959
19391	0.604251223	0.405919894	0.743573733	0.474985329	0.536421655
19393	0.65363472	0.485652088	0.635515371	0.507817138	0.472788443
19394	0.554446991	0.417064629	0.638173565	0.475644699	0.444762814
19395	0.97430161	0.743586607	0.813141409	0.790614391	0.829580269

19396	0.528158153	0.366774542	0.607094452	0.819848975	0.627831715
19398	0.608798632	0.311993406	0.625948507	0.465311169	0.532902871
19399	0.521641021	0.327571305	0.55075475	0.314174589	0.503025065
19401	0.681625105	0.685542971	0.591412142	0.569842327	1.123251166
19403	0.760954556	0.546896102	0.700748873	0.805953144	0.78848036
19404	0.571843073	0.148088531	0.608500204	0.451055701	0.635814889
19428	0.645540797	0.29009434	0.698244782	0.416105121	0.631738544
19429	0.702825782	0.464426877	0.745066558	0.721495895	0.664335664
19430	0.433414044	0.168359942	0.600960584	0.484179971	0.597387518
19431	0.610309705	0.367809132	0.617586448	0.43066489	0.622865447
19435	0.494736231	0	0.567954531	0.216767205	0.407909723
19436	0.520605847	0	0.502289539	0.297802198	0.356043956
19438	0.598411024	0.508833922	0.750015517	0.706713781	0.760306243
19439	0.677275832	0.5350759	0.815441136	0.646366658	0.690020659
19440	0.436911849	0.072909547	0.540189112	0.414900889	0.601048075
19443	0.731997588	0.666850771	0.76967274	0.923070854	0.867050655
19446	0.507081207	0.027848101	0.604692194	0.589873418	0.786708861
19449	0.606279207	0.463629097	0.663309287	0.671995737	0.772448708
19451	0.378508493	0	0.531872912	0.197558281	0.802478408
19452	0.540133856	0.185346707	0.614493423	0.585259485	0.633667684
19453	0.753055641	0.560163481	0.750959714	0.725387667	0.688183676
19455	0.539437215	0.269650029	0.676375253	0.849684452	0.662650602
19457	0.548628116	0	0.641866014	0.225910064	0.281584582
19461	0	0.453542568	0.582298815	0.602903238	0.603707811
19462	0.548369341	0	0.541832943	0.202780554	0.401397256
19463	0.868855719	0.368537787	0.739317832	0.611967326	0.602555288
19466	0.73397082	0.474067454	0.731621361	0.748386456	0.809684573
19467	0.644100119	0.490998363	0.664719905	0.771119224	0.809895505
19468	0.625260274	0.026927672	0.606356164	0.290138774	0.244554728
19469	0.527952167	0.358980957	0.64044416	0.369531652	0.569994853
19471	0.59022183	0	0.611190818	0.16558993	0.379722878
19472	0.537404199	0.304520616	0.550774742	0.549925484	0.732240437
19473	0.424425826	0	0.410980779	0	0.912191827
19474	0.499489602	0.314528594	0.652113046	0.098531685	0.242916023
19648	0.608688888	0.449929304	0.626810778	0.511082244	0.627374752
19649	0.673780393	0.516435354	0.705962521	0.771658145	0.929827124
19651	0.763938883	0.558191048	0.763460619	0.615228426	0.689155515
19654	0.561120296	0.064135454	0.575901974	0.119548486	0.412006157
19657	0.718432416	0.546866953	0.738430671	0.884291845	0.825236052
19662	0.597215207	0.417430859	0.674982979	0.630458515	0.610917031
19678	0.772847682	0.665784531	0.774578082	0.916595527	0.861960048
19681	0.635016934	0.427510002	0.812722721	0.544818061	0.622975805
19684	0.668460332	0.517933804	0.672272998	0.602825093	0.656418779

19716	0.591899818	0	0.583201444	0.193371932	0.397006548
19722	0.669128912	0.482497828	0.640594222	0.759926414	0.707496551
19723	0	0.453570804	0	0.679935219	0.677958585
19725	0.621407405	0.512241055	0.646577886	0.718560368	0.684871312
19726	0.673159298	0.46105919	0.67466796	0.562911042	0.516744548
19728	0.752521465	0.459302647	0.791908815	0.596397193	0.611979886
19731	0.639518821	0.478710211	0.718976632	0.850029856	0.70139176
19749	0.722591808	0.538153846	0.85489693	0.743846154	0.791384615
19750	0.686160949	0.383919891	0.740877243	0.631278761	0.568442543
19755	0.658335784	0.362576228	0.732407135	0.560072751	0.575906708
19756	0.710640263	0.687634371	0.725448877	0.807476247	0.91545877
19758	0.802454745	0.504230118	0.728743829	0.560067682	0.644331641
19761	0.688866207	0.40240804	0.610240009	0.567417026	0.647055733
19771	0.733201913	0.570229035	0.625199228	0.566227177	0.637546004
19773	0.663162122	0.327827592	0.783046764	0.646531383	0.742698338
19776	0.916456785	0.525328982	0.859608458	0.721542502	0.670822014
19777	0.882590389	0.590370084	0.918716423	0.808166272	0.705758792
19779	0.62864547	0.493081569	0.655197847	0.506689091	0.517391637
19782	0.560852032	0.534745375	0.665184572	0.543243773	0.520494215
19783	0.539633704	0.63638985	0.660784257	0.692906574	0.673226644
19785	0.640520167	0.457618481	0.79732023	0.676065908	0.627229489
19786	0.672051879	0.477626883	0.688546445	0.597545051	0.642595978
19794	0.566692702	0.456479691	0.587665249	0.53997421	0.428970557
19795	0.701549955	0.458099397	0.743922837	0.673008942	0.637970472
20503	0.587565272	0.552127431	0.654731164	0.797947908	0.824854703
20504	0.68257329	0.534800041	0.798278269	0.539631952	0.533874782
20505	0.678917308	0.506490787	0.758008443	0.67849665	0.634422111
20507	0.61506368	0.414507772	0.742668228	0.707541739	0.704202648
20508	0.619115279	0.455050505	0.667929652	0.576094276	0.535016835
20509	0.750442822	0.631262525	0.782088392	0.779471987	0.746972205
20510	0.661442372	0.406980417	0.667600619	0.585281112	0.537744788
20515	0.558264067	0.446295542	0.608881393	0.626885312	0.703094657
20517	0.675709886	0.469523002	0.721340872	0.772135316	0.747245126
20518	0.696892521	0.479177156	0.734049226	0.647845789	0.549881321
20520	0.708564775	0.488063416	0.806829678	0.684763573	0.607337082
20521	0.772965981	0.596123369	0.698460742	0.809338047	0.745337072
20522	0.627978607	0.441043303	0.76378874	0.516736968	0.49421462
20524	0.646189421	0.455180768	0.747124	0.66371599	0.567275438
20525	0.736199377	0.436300665	0.926978193	0.609847421	0.616587101
20527	0.855782313	0.53125292	0.872216823	0.736989629	0.748108007
20528	0.737447276	0.472135471	0.931665415	0.601013256	0.581303352
20531	0.607626799	0.419089976	0.675798129	0.685939104	0.566769301
20534	0.869490503	0.523220951	0.947788889	0.751024904	0.638499663

20535	0.69401443	0.518581081	0.657294807	0.880255255	0.673986486
20537	0.624354209	0.44793577	0.78820219	0.599462037	0.562171415
20758	0.60389009	0.291725768	0.609138623	0.570212766	0.620330969
20765	0	0.514385251	0.617666632	0.795680099	0.827718097
20810	0.969783313	0.573164201	0.881394792	0.866235295	0.768281614
20812	0.985188546	0.483674776	1.015798884	0.761363636	0.747119078
100 0	0.474827631	0	0.534885738	0.331746032	0.434920635
101 0	0.621686799	0.545918114	0.680491301	0.811702369	0.849113124
102 0	0.588908419	0.458363193	0.648586221	0.577038842	0.629210262
103 0	0.656815383	0.647351582	0.683503836	0.710510724	0.732669748
104 0	0.714446529	0.65551654	0.770731707	0.738865274	0.708187026
105 0	0.582867216	0.597172948	0.60084422	0.900260671	0.790909417
106 0	0.682286142	0.534089954	0.683263659	0.646574191	0.555947877
107 0	0.638152102	0.470780068	0.647094431	0.637620663	0.579311244
108 0	0.53717722	0.185864978	0.603537319	0.473295784	0.580295678
109 0	0.647727715	0.303142329	0.566026714	0.494762785	0.661737523
110 0	0.570038627	0.471849234	0.704759128	0.802355713	0.60753828
111 0	0.572225481	0.492319579	0.657703416	0.697379488	0.728081327
112 0	0.614281206	0.39206244	0.561717728	0.772283365	0.74871452
113 0	0.571901345	0.255520505	0.591513969	0.593410445	0.638626008
114 0	0.639110901	0.255175009	0.675242374	0.508844562	0.608957471
115 0	0.587050514	0.418083856	0.604831658	0.632995298	0.550639714
116 0	0.547435565	0.543921293	0.567219272	0.508315765	0.460763645
117 0	0.681285197	0.443837209	0.702233588	0.549883721	0.502209302
118 0	0.50117013	0	0.549960996	0.611692845	0.629144852
119 0	0.618375247	0.51828125	0.608126549	0.798161171	0.67328125
120 0	0.548909465	0.271324635	0.558836416	0.447606609	0.595588413
121 0	0.632140524	0.203464394	0.605973724	0.456420126	0.459444597
122 0	0.543772696	0.04387292	0.480861714	0.295007564	0.712556732
123 0	0.527775695	0.175645756	0.542356998	0.650184502	0.619926199
124 0	0.696044251	0.504264467	0.771848158	0.703319941	0.610277014
125 0	0.58425419	0.505077031	0.638162093	0.639880952	0.538340336
126 0	0.741506401	0.548370686	0.745367548	1.150828714	0.857257611
127 0	0.715253677	0.478202995	0.730237426	0.627870216	0.684442596
128 N	0	0.459582168	0	0.681059248	0.827788526
129 0	0.668573561	0.538174177	0.59041164	0.729161802	0.656549148
130 0	0.740565932	0.566743531	0.782437687	0.678708686	0.671449312
131 0	0.61272537	0.533598266	0.678666291	0.719894027	0.725794798
132 0	0.652073949	0.481057616	0.748850413	0.590370955	0.571132597
133 0	0.554557271	0.544084998	0.613276173	0.783539486	0.746907707
134 0	0.737344657	0.604271645	0.738939268	0.88281926	0.879784445
136 0	0.630538414	0.485619206	0.658848302	0.734490135	0.75136677
137 0	0.495719773	0.441597338	0.567192355	0.735440932	0.721464226

138 0	0.626666382	0.373922648	0.651106065	0.655831474	0.698369157
139 0	0.674990191	0.509429429	0.716551763	0.80954955	0.821441441
140 0	0.940543903	0.420154102	1.009924162	0.673289016	0.712720955
141 0	0.651394592	0.486823289	0.696927651	0.916036772	0.86741573
142 0	0.841544145	0.513391821	0.63989785	0.69609618	0.883071067
143 0	0.616775634	0.408063198	0.630106841	0.64233179	0.683192591
145 0	0.729570345	0.562877979	0.637061759	0.671824973	0.829242263
148 0	0.673608551	0.553380783	0.647207888	0.792170819	0.753736655
149 0	0.687391835	0.503370215	0.727684988	0.822713977	0.822332443
150 0	0.642857143	0.427027027	0.758535036	0.444698545	0.446985447
151 0	0.577657452	0.238033495	0.594186378	0.726580227	0.734683955
152 N	0	0.590017303	0	0.770736509	0.793692293
155 0	0.697966919	0.455527847	0.695511716	0.647963425	0.717788861
156 0	0.608559127	0.432411067	0.661357677	0.832147563	0.766798419
157 0	0.692173534	0.548648649	0.764006976	0.885135135	0.737714988
158 0	0.575220388	0.326574946	0.604103222	0.604634323	0.536929761
159 0	0.772752809	0.467425432	0.813623596	0.731161695	0.821428571
160 0	0.685405891	0.630798874	0.723627373	0.803517613	0.82970209
231 0	0.619856235	0.49157003	0.599819426	0.728760589	0.713792253
232 0	0.653463945	0.615450443	0.713145154	0.845806753	0.90508791
233 0	0.602860866	0.442486383	0.703518679	0.597564883	0.633771227
234 0	0.595764185	0.395857757	0.751688531	0.500586166	0.513872606
235 0	0.591927294	0.311449963	0.693369459	0.550127831	0.498995617
236 0	0.618688593	0.446013124	0.656980023	0.650361238	0.690594552
237 0	0.657101953	0.403956605	0.641841055	0.771818342	0.717081472
238 0	0.568913771	0.338314486	0.585276463	0.370355412	0.372778675
239 0	0.684754522	0.488757278	0.774706255	0.726962457	0.699056414
240 0	0.624422442	0.690453686	0.651797811	1.030245747	1.052693762
242 0	0.698517705	0.680331444	0.657589898	0.85662887	0.889991278
243 0	0.661387452	0.649856105	0.768298183	0.905029464	1.004933534
244 0	0.641496707	0.49156939	0.621339576	0.530912235	0.559014267
245 0	0.633553429	0.635172999	0.703056129	0.708504599	0.771674143
246 0	0.585263806	0.474978796	0.523857868	0.57902177	0.706813684
247 0	0.652569369	0.333723653	0.573448459	0.532786885	0.798302108
249 0	0.609615651	0.22980063	0.574927154	0.125918153	0.44910808
250 0	0.641211638	0.541124633	0.645237146	0.63701217	0.75472094
251 0	0.53663404	0.314144737	0.521761855	0.030153509	0.233552632
252 0	0.694884075	0.453690037	0.672555425	0.669372694	0.631549815
253 0	0.705905564	0.434260775	0.675829716	0.593289689	0.698781851
254 0	0.608814103	0.457110179	0.550520833	0.388867709	0.558520778
255 0	0.607658628	0.425045078	0.586257756	1.288705997	1.234036138
256 0	0.58173924	0.432349766	0.640762648	0.761099313	0.743928179
257 0	0.605266002	0.462601995	0.689514822	0.739202376	0.770398912

258 0	0.530030504	0.32593985	0.634620104	0.284210526	0.6
259 0	0.596002988	0.336596233	0.549943967	0.458718693	0.55714575
260 0	0.523982454	0.386693017	0.553538041	0.578392622	0.655467721
261 0	0.66760903	0.392083333	0.63172993	0.501041667	0.570625
262 N	0	0.421440482	0.63005371	0.549090727	0.585008757
263 0	0.629009332	0.284649776	0.530066127	0.258320914	0.420765027
264 0	0.565417105	0.364113326	0.588384133	0.390346275	0.51381602
265 0	0.658123721	0.46703221	0.647456257	0.664806801	0.707726067
96 0.	7.06427E+14	0.542093387	0.759646545	0.71003298	0.712549905
97 0.	6.90029E+14	0.574014425	0.70177373	0.71360549	0.632413697
98 0.	6.37396E+14	0.440829038	0.724916988	0.651632302	0.609428694
99 0.	7.82804E+14	0.708369263	0.853407509	0.802745042	0.815823513
