# Supplementary material for: Pharmacogenomic Analyses Implicate B Cell Developmental Status and MKL1 as Determinants of Sensitivity toward Anti-CD20 Monoclonal Antibody Therapy
Source: Cells. 2023 Jun 7;12(12):1574. doi: 10.3390/cells12121574 (PMC10297299; doi:10.3390/cells12121574)
Supplement: Supplementary file 1 [file cells-12-01574-s001.zip › Table S3-Gene expression results.pdf]

Table S2: Gene expression levels that are significantly correlated with sensitivity.

The significant gene results (p <0.05, corrected Bonferroni p-value) are shown for each respective antibody in bold. The p-value is also shown when p > 0.05 to 0.25 if p is <0.05 for another antibody. Data is sorted based on drug then level of significance.

	Ensembl ID	gene name	ofat p-value n=221	obin p-value n=8	rtx p-value n=2
**rituximab**	ENSG00000151079.6	KCNA6	0.0103	0.1672	**0.0372**
	ENSG00000102870.4	ZNF629	0.0016	0.1877	**0.0372**
**obinutuzumab**	ENSG00000146416.10	AIG1	0.0103	**0.0016**	---
	ENSG00000018408.10	WWTR1	0.0436	**0.0102**	---
	ENSG00000223478.1	RP11-545E17.3	0.0033	**0.0102**	---
	ENSG00000261609.1	GAN	0.0072	**0.0102**	---
	ENSG00000223886.3	RP11-251G23.2	0.1485	**0.0184**	---
	ENSG00000196083.5	IL1RAP	0.0084	**0.0184**	---
	ENSG00000136444.5	RSAD1	0.0698	**0.0184**	---
	ENSG00000150593.11	PDCD4	0.2059	**0.0388**	---
**ofatumumab**	ENSG00000140682.14	**TGFB1I1**	**0.0003**	---	---
	ENSG00000102870.4	ZNF629	**0.0016**	0.1877	0.0372
	ENSG00000262026.1	ENSG00000262026.1	**0.0017**	0.2180	0.0757
	ENSG00000075234.12	WSB2	**0.0025**	---	---
	ENSG00000145936.3	KCNMB1	**0.0025**	0.1620	---
	ENSG00000141391.8	PRELID3A	**0.0025**	---	---
	ENSG00000160233.5	LRRC3	**0.0025**	0.2323	0.2059
	ENSG00000155254.7	MARVELD1	**0.0025**	0.1240	---
	ENSG00000176871.4	RP11-545E17.3	**0.0033**	0.0102	---
	ENSG00000188290.6	HES4	**0.0039**	---	---
	ENSG00000102996.4	MMP15	**0.0042**	---	---
	ENSG00000102931.3	ARL2BP	**0.0069**	---	---
	ENSG00000223478.1	FCMR	**0.0072**	0.2198	---
	ENSG00000146416.10	DPF3	**0.0072**	---	---
	ENSG00000241399.2	CD302	**0.0072**	0.0941	---
	ENSG00000143878.8	RHOB	**0.0072**	---	---
	ENSG00000261609.1	GAN	**0.0072**	0.0102	---
	ENSG00000110080.13	ST3GAL4	**0.0072**	---	---
	ENSG00000188997.3	KCTD21	**0.0084**		
	ENSG00000082781.6	GNAS	**0.0084**		
	ENSG00000186314.7	PRELID2	**0.0084**		
	ENSG00000168026.11	TTC21A	**0.0084**	0.2050	
	ENSG00000181649.5	PHLDA2	**0.0084**		
	ENSG00000196083.5	IL1RAP	**0.0084**	0.0184	

ENSG00000178773.10	CPNE7	**0.0084**	0.2353	
ENSG00000110237.3	ARHGEF17	**0.0084**		
ENSG00000107738.12	C10orf54	**0.0085**	0.1786	
ENSG00000171368.10	TPPP	**0.0089**	0.2353	
ENSG00000120029.7	C10orf76	**0.0093**		
ENSG00000224407.1	RP5-956O18.3	**0.0093**		
ENSG00000198682.7	PAPSS2	**0.0093**	0.2435	
ENSG00000114853.8	ZBTB47	**0.0096**		
ENSG00000170634.7	AIG1	**0.0103**	0.0016	
ENSG00000162894.7	SERPINA1	**0.0103**	0.2300	0.1211
ENSG00000108771.6	DHX58	**0.0103**		
ENSG00000132205.5	EMILIN2	**0.0103**		
ENSG00000151079.6	KCNA6	**0.0103**	0.1672	0.0372
ENSG00000198003.5	CCDC151	**0.0103**		
ENSG00000136040.4	RP1-148H17.1	**0.0104**		
ENSG00000227677.1	ENSG00000227677.1	**0.0104**		
ENSG00000179431.5	FJX1	**0.0104**		
ENSG00000087460.15	ACYP2	**0.0115**		
ENSG00000196352.8	CD55	**0.0115**		
ENSG00000164683.11	HEY1	**0.0115**		
ENSG00000197249.8	CYB561A3	**0.0119**		
ENSG00000165233.13	CARD19	**0.0119**		
ENSG00000143641.7	GALNT2	**0.0119**		
ENSG00000136997.9	MYC	**0.0119**		
ENSG00000160285.9	LSS	**0.0121**		
ENSG00000162144.4	SLC6A4	**0.0128**		
ENSG00000197077.7	TRIM32	**0.0138**		
ENSG00000246082.2	NUDT16P1	**0.0138**		
ENSG00000149591.11	TAGLN	**0.0144**	0.1187	
ENSG00000164086.8	DUSP7	**0.0144**		
ENSG00000139112.6	PLXNC1	**0.0144**		
ENSG00000108576.4	KIAA1671	**0.0144**		
ENSG00000104388.9	RAB2A	**0.0144**		
ENSG00000010803.12	SCMH1	**0.0146**		
ENSG00000182022.12	CHST15	**0.0152**		
ENSG00000138002.10	IFT172	**0.0152**		
ENSG00000120896.9	SORBS3	**0.0155**		
ENSG00000076706.7	MCAM	**0.0155**		
ENSG00000178403.3	NEUROG2	**0.0156**		
ENSG00000137177.13	KIF13A	**0.0158**		
ENSG00000119401.9	ADO	**0.0170**		
ENSG00000197355.6	UAP1L1	**0.0170**		
ENSG00000096433.5	ITPR3	**0.0170**		

ENSG00000205683.5	GABARAPL1	**0.0177**		
ENSG00000187824.3	TMEM220	**0.0177**		
ENSG00000237513.1	RP11-325F22.2	**0.0177**		
ENSG00000176014.6	TUBB6	**0.0186**		
ENSG00000082146.6	ITGB5	**0.0194**		
ENSG00000181915.3	TK2	**0.0202**		
ENSG00000257607.1	RP11-449P15.1	**0.0202**		
ENSG00000171813.10	PWWP2B	**0.0202**		
ENSG00000143537.8	ADAM15	**0.0202**		
ENSG00000114993.11	RTKN	**0.0202**		
ENSG00000170190.10	SLC16A5	**0.0202**		
ENSG00000165912.10	PACSIN3	**0.0202**		
ENSG00000163132.6	MSX1	**0.0202**		
ENSG00000177096.4	FAM109B	**0.0203**		
ENSG00000197701.7	ENSG00000197701.7	**0.0206**		
ENSG00000158747.9	NBL1	**0.0206**		
ENSG00000106624.4	AEBP1	**0.0209**		
ENSG00000144283.15	PKP4	**0.0209**		
ENSG00000224565.1	PRKACA	**0.0213**		
ENSG00000100311.12	PDGFB	**0.0215**		
ENSG00000166311.5	STRADB	**0.0231**		
ENSG00000131037.8	EPS8L1	**0.0236**		
ENSG00000184014.3	GPS2	**0.0244**		
ENSG00000172716.11	SLFN11	**0.0246**		
ENSG00000142733.8	MAP3K6	**0.0246**		
ENSG00000115107.14	STEAP3	**0.0246**		
ENSG00000072062.7	FHL2	**0.0257**	0.1016	
ENSG00000235105.1	UBE2Q1	**0.0257**	0.0609	
ENSG00000160584.11	RAB11FIP1	**0.0257**		
ENSG00000132522.10	BCAR3	**0.0257**		
ENSG00000129195.9	FAM64A	**0.0257**		
ENSG00000128917.5	DLL4	**0.0257**		
ENSG00000118257.11	NRP2	**0.0257**	0.0609	
ENSG00000188157.8	AGRN	**0.0257**		
ENSG00000020577.9	SAMD4A	**0.0257**		
ENSG00000139832.3	RAB20	**0.0257**		
ENSG00000205277.5	MUC12	**0.0257**		
ENSG00000177425.6	PAWR	**0.0257**		
ENSG00000105186.7	ANKRD27	**0.0257**		
ENSG00000157782.5	CABP1	**0.0260**		
ENSG00000213205.3	RCAN1	**0.0261**		
ENSG00000168916.10	TP73	**0.0269**		
ENSG00000167771.5	RCOR2	**0.0271**		

ENSG00000160447.6	PKN3	**0.0271**		
ENSG00000187678.7	SPRY4	**0.0274**		
ENSG00000250073.2	RP11-677M14.3	**0.0274**		
ENSG00000116132.7	PRRX1	**0.0274**		
ENSG00000092621.6	PHGDH	**0.0274**		
ENSG00000139926.11	RP11-329A14.1	**0.0283**		
ENSG00000240024.1	SMPD1	**0.0285**		
ENSG00000153395.5	LPCAT1	**0.0285**		
ENSG00000198598.2	MMP17	**0.0285**		
ENSG00000213362.3	FTH1P12	**0.0285**		
ENSG00000138463.8	PRSS16	**0.0286**		
ENSG00000166548.10	ZNF658B	**0.0286**		
ENSG00000142102.11	PGGHG	**0.0286**		
ENSG00000074660.11	SCARF1	**0.0286**		
ENSG00000149564.7	ESAM	**0.0286**		
ENSG00000004399.8	PLXND1	**0.0288**		
ENSG00000101670.5	LIPG	**0.0295**		
ENSG00000100968.9	NFATC4	**0.0295**		
ENSG00000115641.13	ENSG00000198566.7	**0.0302**		
ENSG00000140854.8	KATNB1	**0.0302**		
ENSG00000141401.6	IMPA2	**0.0302**		
ENSG00000124006.8	OBSL1	**0.0315**		
ENSG00000100344.6	PNPLA3	**0.0328**		
ENSG00000079263.11	FCRL3	**0.0330**		
ENSG00000136098.10	NEK3	**0.0330**		
ENSG00000253276.1	DENND5A	**0.0333**		
ENSG00000078900.9	RPS4XP16	**0.0333**	0.0856	
ENSG00000198873.8	GRK5	**0.0333**	0.0923	
ENSG00000176659.5	C20orf197	**0.0333**		
ENSG00000224892.1	ITGAX	**0.0337**		
ENSG00000170412.11	GPRC5C	**0.0342**		
ENSG00000009790.9	APLF	**0.0343**		
ENSG00000159200.12	IL24	**0.0343**		
ENSG00000261658.1	ENSG00000261658.1	**0.0343**		
ENSG00000056661.8	ENSG00000056661.8	**0.0343**		
ENSG00000136010.9	ALDH1L2	**0.0344**		
ENSG00000197905.4	TEAD4	**0.0352**		
ENSG00000169418.7	NPR1	**0.0358**		
ENSG00000105655.10	ISYNA1	**0.0374**		
ENSG00000230055.1	ENSG00000230055.1	**0.0377**		
ENSG00000253228.1	SIK3	**0.0387**		
ENSG00000162692.5	LINC00888	**0.0387**		
ENSG00000160714.5	TRAF3IP3	**0.0387**		

ENSG00000140678.12	CMTM1	**0.0387**		
ENSG00000130477.9	UNC13A	**0.0387**		
ENSG00000148180.9	GSN	**0.0387**		
ENSG00000131584.13	ACAP3	**0.0387**	0.0746	
ENSG00000105514.2	RAB3D	**0.0387**		
ENSG00000128408.7	RIBC2	**0.0387**		
ENSG00000008256.11	CYTH3	**0.0387**	0.0609	
ENSG00000147454.8	SLC25A37	**0.0387**		
ENSG00000018408.10	SP140	**0.0393**		
ENSG00000143420.12	STRADBP1	**0.0393**		
ENSG00000163947.7	CAMP	**0.0393**		
ENSG00000158106.8	RHPN1	**0.0393**		
ENSG00000169621.5	TTC38	**0.0395**		
ENSG00000132434.5	NRBF2P4	**0.0396**		
ENSG00000178814.11	OPLAH	**0.0396**		
ENSG00000254102.1	RP11-21C4.1	**0.0399**		
ENSG00000162892.11	VCAM1	**0.0409**		
ENSG00000064651.9	SLC12A2	**0.0409**		
ENSG00000068383.12	INPP5A	**0.0409**		
ENSG00000162714.7	ZNF496	**0.0422**		
ENSG00000213523.4	DIRC2	**0.0427**		
ENSG00000087903.7	RFX2	**0.0427**		
ENSG00000198093.5	ZNF649	**0.0427**	0.1014	0.2059
ENSG00000154760.9	SLFN13	**0.0427**		
ENSG00000112812.10	WWTR1	**0.0436**		
ENSG00000105613.3	MAST1	**0.0436**		
ENSG00000043462.7	LCP2	**0.0436**		
ENSG00000249509.1	RP11-402J6.1	**0.0436**		
ENSG00000156675.10	CCDC71L	**0.0438**		
ENSG00000137936.11	ENSA	**0.0439**		
ENSG00000165646.7	SLC18A2	**0.0439**		
ENSG00000196155.7	PLEKHG4	**0.0439**		
ENSG00000067182.3	TNFRSF1A	**0.0440**		
ENSG00000112144.11	ICK	**0.0440**		
ENSG00000160961.6	ZNF333	**0.0440**		
ENSG00000260528.1	FAM157C	**0.0440**		
ENSG00000196267.6	ZNF836	**0.0440**		
ENSG00000004660.10	CAMKK1	**0.0444**		
ENSG00000115756.8	HPCAL1	**0.0447**		
ENSG00000089505.12	LANCL2	**0.0449**		
ENSG00000102471.9	SRA1	**0.0449**		
ENSG00000140368.8	PSTPIP1	**0.0449**		
ENSG00000236233.2	ENSG00000236233.2	**0.0449**		

	ENSG00000164849.6	GPR146	**0.0449**		
	ENSG00000187961.7	KLHL17	**0.0449**		
	ENSG00000179818.6	PCBP1-AS1	**0.0449**		
	ENSG00000237198.2	ENSG00000237198.2	**0.0452**		
	ENSG00000198416.7	ZNF608	**0.0452**		
	ENSG00000160856.14	FRMD6	**0.0452**		
	ENSG00000127418.9	FGFRL1	**0.0454**		
	ENSG00000109062.4	SLC9A3R1	**0.0456**		
	ENSG00000198566.7	ARHGEF3	**0.0459**		
	ENSG00000164047.3	IRF6	**0.0462**		
	ENSG00000162572.14	SCNN1D	**0.0466**		
	ENSG00000134504.7	NDFIP2	**0.0470**		
	ENSG00000117595.6	BORCS5	**0.0473**		
	ENSG00000110328.5	GALNT18	**0.0473**		
	ENSG00000138080.8	EMILIN1	**0.0483**		
	ENSG00000086288.7	ENSG00000196409.6	**0.0487**		
	ENSG00000165714.6	KCTD1	**0.0488**		
	ENSG00000259820.1	AC083843.1	**0.0489**		
	ENSG00000198429.5	ZNF69	**0.0494**		
	ENSG00000196409.6	NME8	**0.0497**		
	ENSG00000158270.9	COLEC12	**0.0497**		
	ENSG00000089091.10	DZANK1	**0.0497**		
	ENSG00000237493.2	RP11-603J24.7	**0.0499**		
	ENSG00000100979.10	PLTP	**0.0499**		
	ENSG00000206082.4	ENSG00000206082.4	**0.0499**	0.0746	
