# Supplementary material for: Pharmacogenomic Analyses Implicate B Cell Developmental Status and MKL1 as Determinants of Sensitivity toward Anti-CD20 Monoclonal Antibody Therapy
Source: Cells. 2023 Jun 7;12(12):1574. doi: 10.3390/cells12121574 (PMC10297299; doi:10.3390/cells12121574)
Supplement: Supplementary file 1 [file cells-12-01574-s001.zip › Table S4-supervised gene expression list.pdf]

Table S3: Gene list for supervised gene expression analysis

Ensembl ID	gene name	Ensembl ID	gene name	Ensembl ID	gene name
ENSG00000183044	ABAT	ENSG00000070831	CDC42	ENSG00000196092	PAX5
ENSG00000154262	ABCA6	ENSG00000111666	CHPT1	ENSG00000143393	PI4KB
ENSG00000097021	ACOT7	ENSG00000141367	CLTC	ENSG00000105647	PIK3R2
ENSG00000124942	AHNAK	ENSG00000091317	CMTM6	ENSG00000127838	PNKD
ENSG00000111732	AICDA	ENSG00000055130	CUL1	ENSG00000105171	POP4
ENSG00000111732	AICDA	ENSG00000107562	CXCL12	ENSG00000184271	POU6F1
ENSG00000155085	AK9	ENSG00000205795	CYS1	ENSG00000135362	PRR5L
ENSG00000135046	anxa11	ENSG00000184545	DUSP8	ENSG00000135069	PSAT1
ENSG00000122359	ANXA11	ENSG00000004478	FKBP4	ENSG00000125637	PSD4
ENSG00000182718	ANXA2	ENSG00000070404	FSTL3	ENSG00000144566	RAB5A
ENSG00000164111	ANXA5	ENSG00000139112	GABARAPL1	ENSG00000113522	RAD50
ENSG00000197043	ANXA6	ENSG00000178234	GALNT11	ENSG00000197747	S100A10
ENSG00000138279	ANXA7	ENSG00000139436	GIT2	ENSG00000163191	S100A11
ENSG00000134982	APC	ENSG00000139436	GIT2	ENSG00000155307	SAMSN1
ENSG00000143761	ARF1	ENSG00000204257.8	HLA-DMA	ENSG00000103496	SCAMP5
ENSG00000029153	ARNTL2	ENSG00000242574.3	HLA-DMB	ENSG00000138468	SENP7
ENSG00000095585	BLNK	ENSG00000241106.2	HLA-DOB	ENSG00000163082	SGPP2
ENSG00000163689	C3orf67	ENSG00000223865.4	HLA-DPB1	ENSG00000160584	SIK3
ENSG00000134072	CAMK1	ENSG00000196735.6	HLA-DQA1	ENSG00000108854	SMURF2
ENSG00000014216	CAPN1	ENSG00000196126.6	HLA-DRB1	ENSG00000198369	SPRED2
ENSG00000162909	CAPN2	ENSG00000211898	IGHD	ENSG00000128578	STRIP2
ENSG00000092529	CAPN3	ENSG00000211896	IGHG1	ENSG00000135604	STX11
ENSG00000130940	CASZ1	ENSG00000211893	IGHG2	ENSG00000099365	STX1B
ENSG00000105974	CAV1	ENSG00000211897	IGHG3	ENSG00000111450	STX2
ENSG00000114423	CBLB	ENSG00000211899	IGHM	ENSG00000166900	STX3
ENSG00000119242	CCDC92	ENSG00000211943.2	IGHV3-15	ENSG00000103496	STX4
ENSG00000102970	CCL17	ENSG00000211967.2	IGHV3-53	ENSG00000079950	STX7
ENSG00000151882	CCL28	ENSG00000211966.2	IGHV5-51	ENSG00000170310	STX8
ENSG00000151882	CCL28	ENSG00000243238.1	IGKV2-30	ENSG00000076944	STXBP2
ENSG00000150637	CD226	ENSG00000242534.2	IGKV2D-28	ENSG00000106799	TGFBR1
ENSG00000139193	CD27	ENSG00000239571.1	IGKV2D-30	ENSG00000120949	TNFRSF8
ENSG00000004468	CD38	ENSG00000253818.1	IGLV1-41	ENSG00000167460	TPM4
ENSG00000101017	CD40	ENSG00000211668.2	IGLV2-11	ENSG00000104447	TRPS1
ENSG00000026508	CD44	ENSG00000211664.2	IGLV2-18	ENSG00000074319	TSG101
ENSG00000026508	CD44	ENSG00000211659.2	IGLV3-25	ENSG00000074319	TSG101
ENSG00000117335	CD46	ENSG00000134460	IL2RA	ENSG00000111647	UHRF1BP1L
ENSG00000196352	CD55	ENSG00000152969	JAKMIP1	ENSG00000118640	VAMP8
ENSG00000085063	CD59	ENSG00000185896	LAMP1	ENSG00000131475	VPS25
ENSG00000110848	CD69	ENSG00000156738	MS4A1	ENSG00000160948	VPS28
ENSG00000019582.10	CD74	ENSG00000118513	MYB	ENSG00000136100	VPS36

ENSG00000105369	CD79A	ENSG00000157483	MYO1E	ENSG00000115085	ZAP70
ENSG00000007312	CD79B	ENSG00000167306	MYO5B	ENSG00000175893	ZDHHC21
ENSG00000114013	CD86	ENSG00000113580	NR3C1	ENSG00000075292	ZNF638
ENSG00000010278	CD9	ENSG00000198836	OPA1	ENSG00000198146	ZNF770
