# Supplementary material for: Pharmacogenomic Analyses Implicate B Cell Developmental Status and MKL1 as Determinants of Sensitivity toward Anti-CD20 Monoclonal Antibody Therapy
Source: Cells. 2023 Jun 7;12(12):1574. doi: 10.3390/cells12121574 (PMC10297299; doi:10.3390/cells12121574)
Supplement: Supplementary file 1 [file cells-12-01574-s001.zip › Table S5-supervised gene expression results.pdf]

Table S4 : Supervised gene expression analysis results for ofatumumab+complement

Results are shown where the corrected p-value is less than 0.05

Gene ID		p value
ENSG00000196352	CD55	0.004
ENSG00000139112	GABARAPL1	0.005
ENSG00000160584	SIK3	0.017
ENSG00000204257	HLA-DMA	0.029
ENSG00000242574	HLA-DMB	0.031
ENSG00000205795	CYS1	0.044
ENSG00000196126	HLA-DRB1	0.044
ENSG00000091317	CMTM6	0.044
ENSG00000198794	SCAMP5	0.044
ENSG00000111450	STX2	0.046
ENSG00000167306	MYO5B	0.048
ENSG00000019582	CD74	0.048
