# Supplementary figures and images for: Pharmacogenomic Analyses Implicate B Cell Developmental Status and MKL1 as Determinants of Sensitivity toward Anti-CD20 Monoclonal Antibody Therapy
Source: Cells. 2023 Jun 7;12(12):1574. doi: 10.3390/cells12121574 (PMC10297299; doi:10.3390/cells12121574)

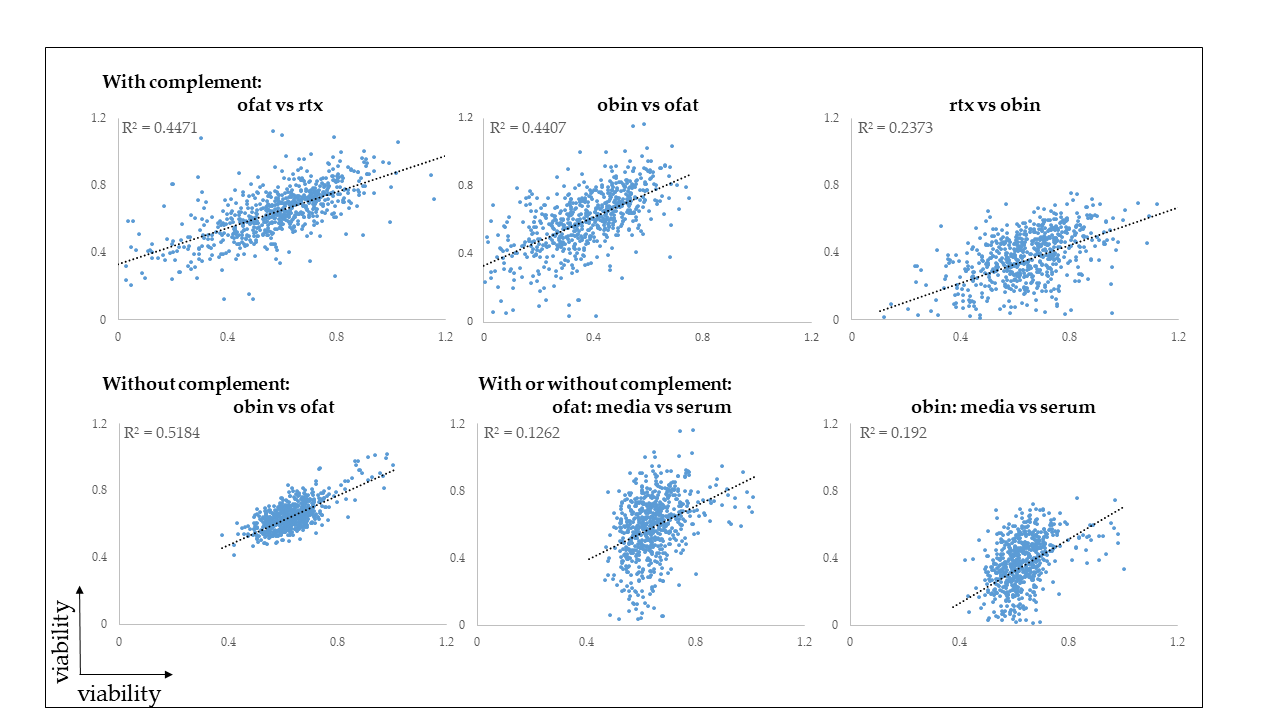

Supplement: Supplementary file 1 [file cells-12-01574-s001.zip › Figure S1-Scatterplots.tif]

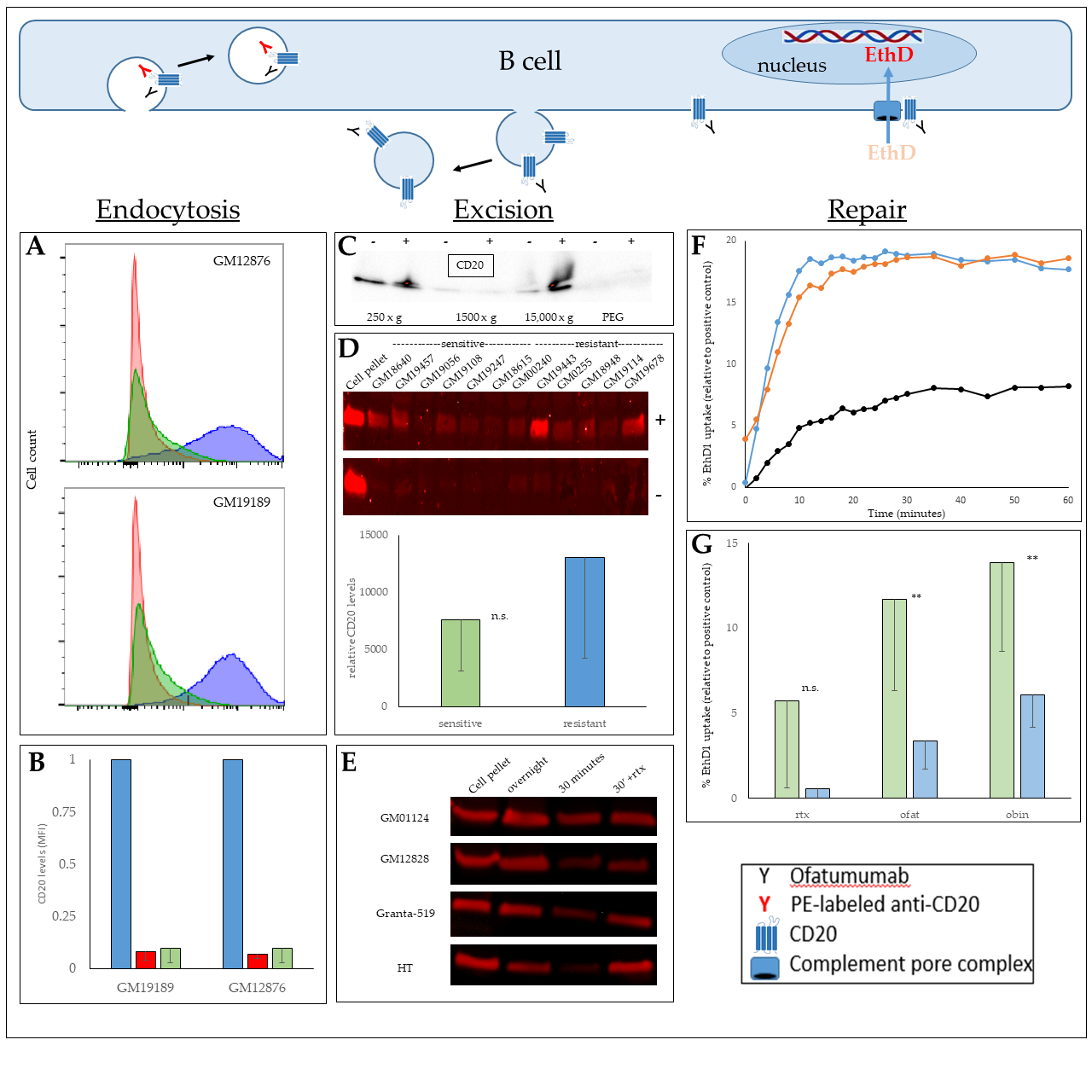

Supplement: Supplementary file 1 [file cells-12-01574-s001.zip › Figure S10-protective mechanisms.tif]

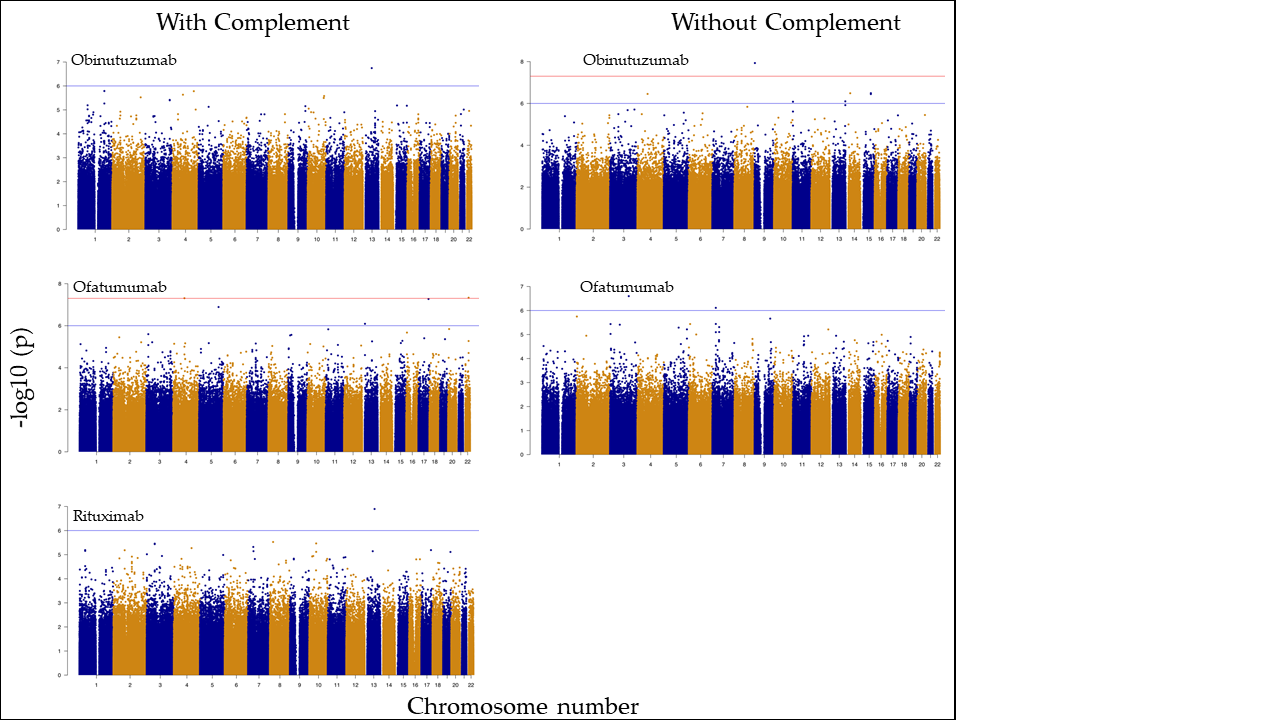

Supplement: Supplementary file 1 [file cells-12-01574-s001.zip › Figure S2-Manhattan plots.tif]

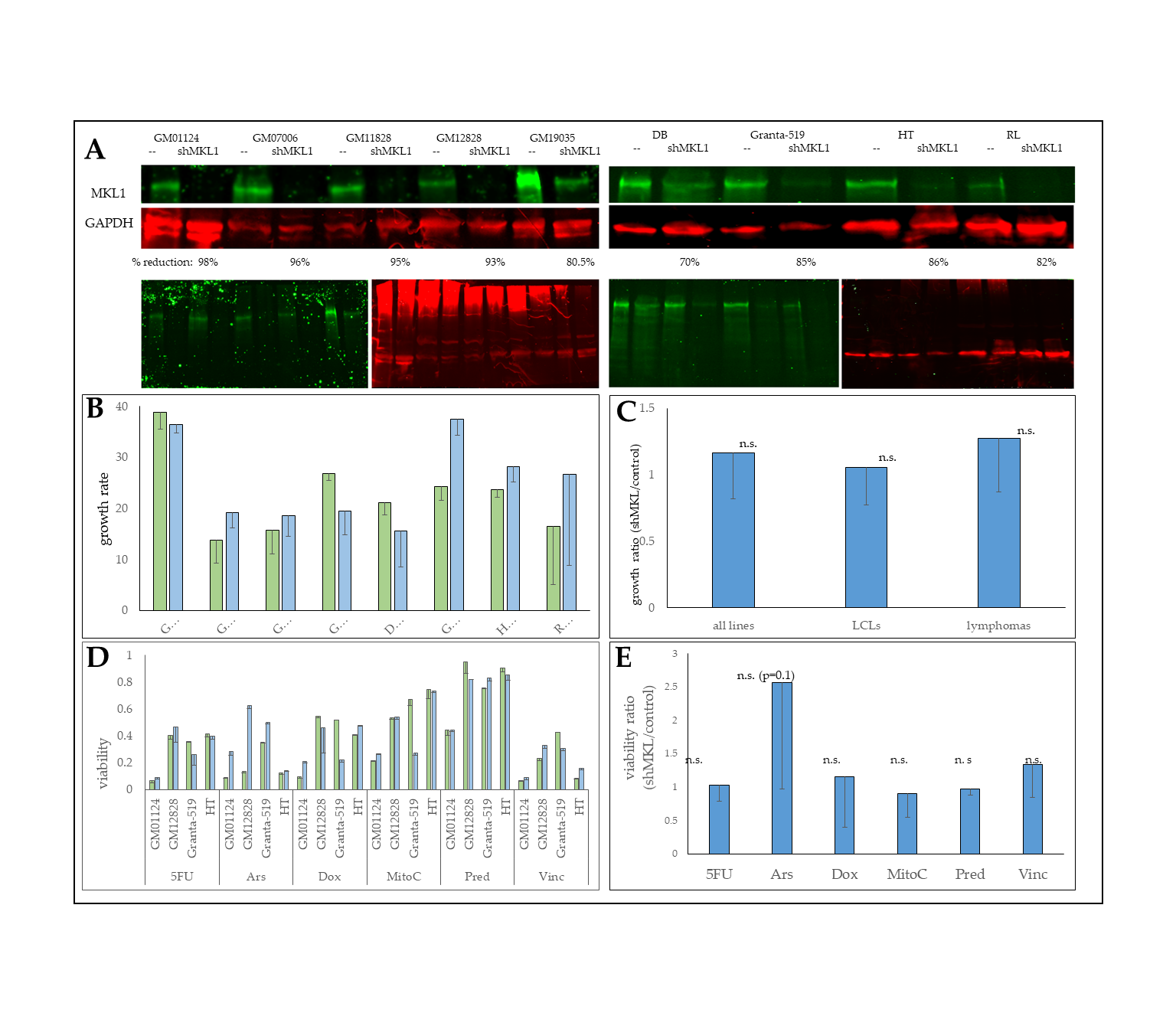

Supplement: Supplementary file 1 [file cells-12-01574-s001.zip › Figure S3-sMKL characterization 6-1-23.tif]

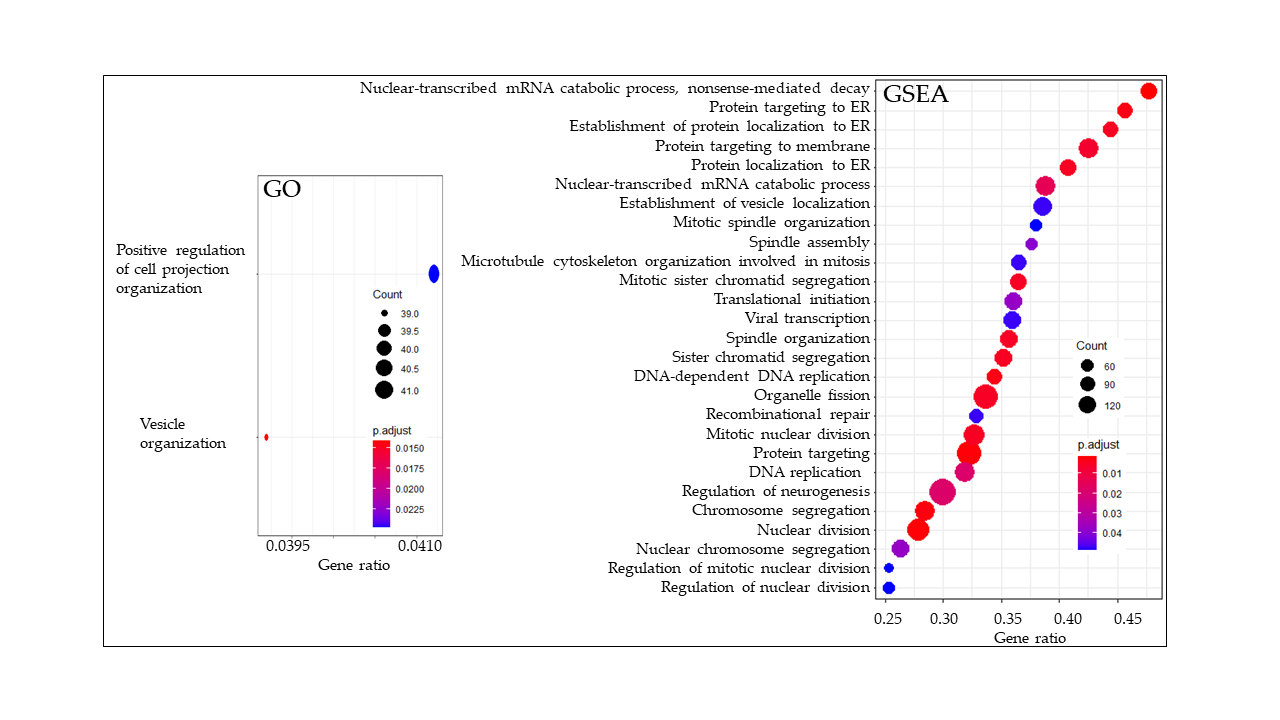

Supplement: Supplementary file 1 [file cells-12-01574-s001.zip › Figure S4-GO and GSEA.tif]

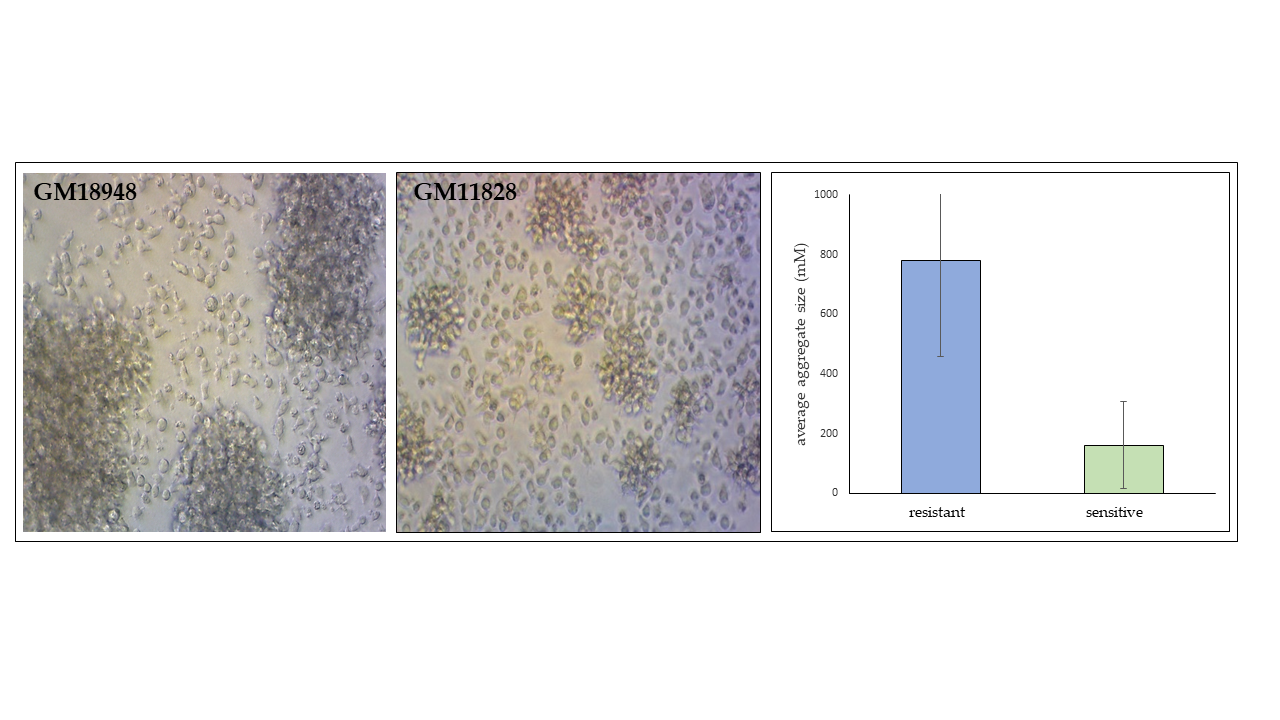

Supplement: Supplementary file 1 [file cells-12-01574-s001.zip › Figure S5-homotypic aggregation.tif]

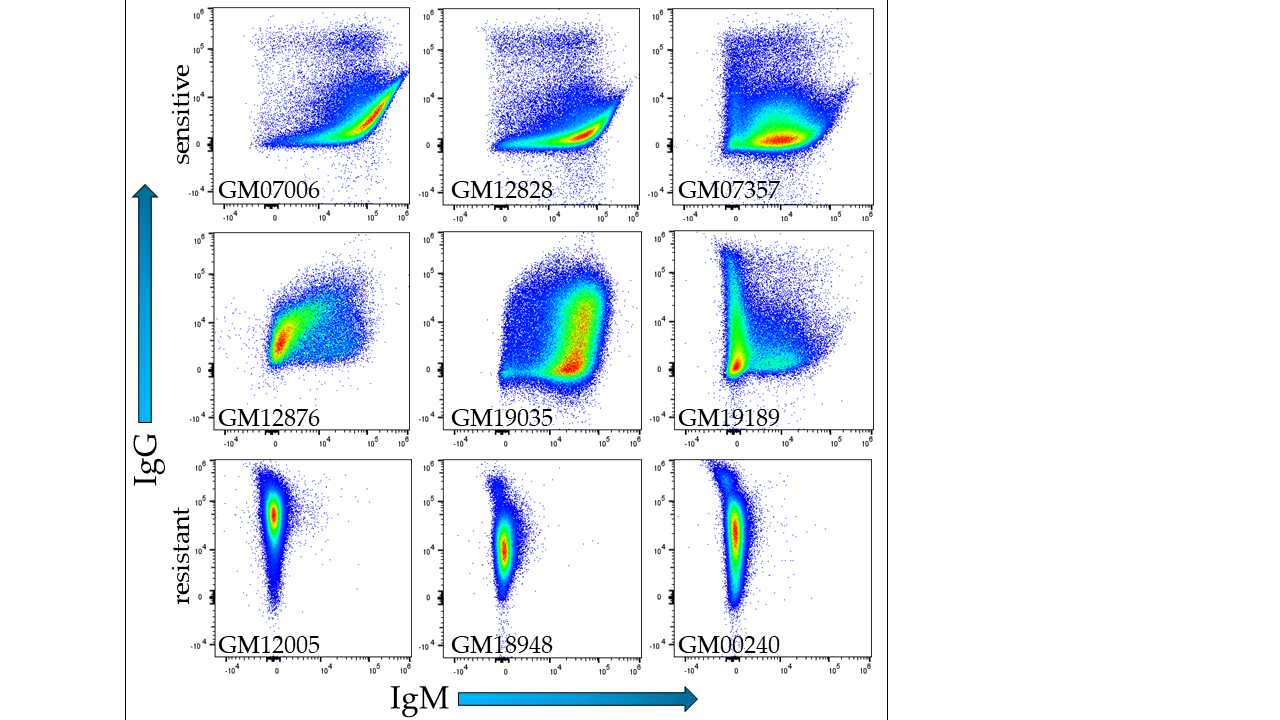

Supplement: Supplementary file 1 [file cells-12-01574-s001.zip › Figure S6-Surface Ig.tif]

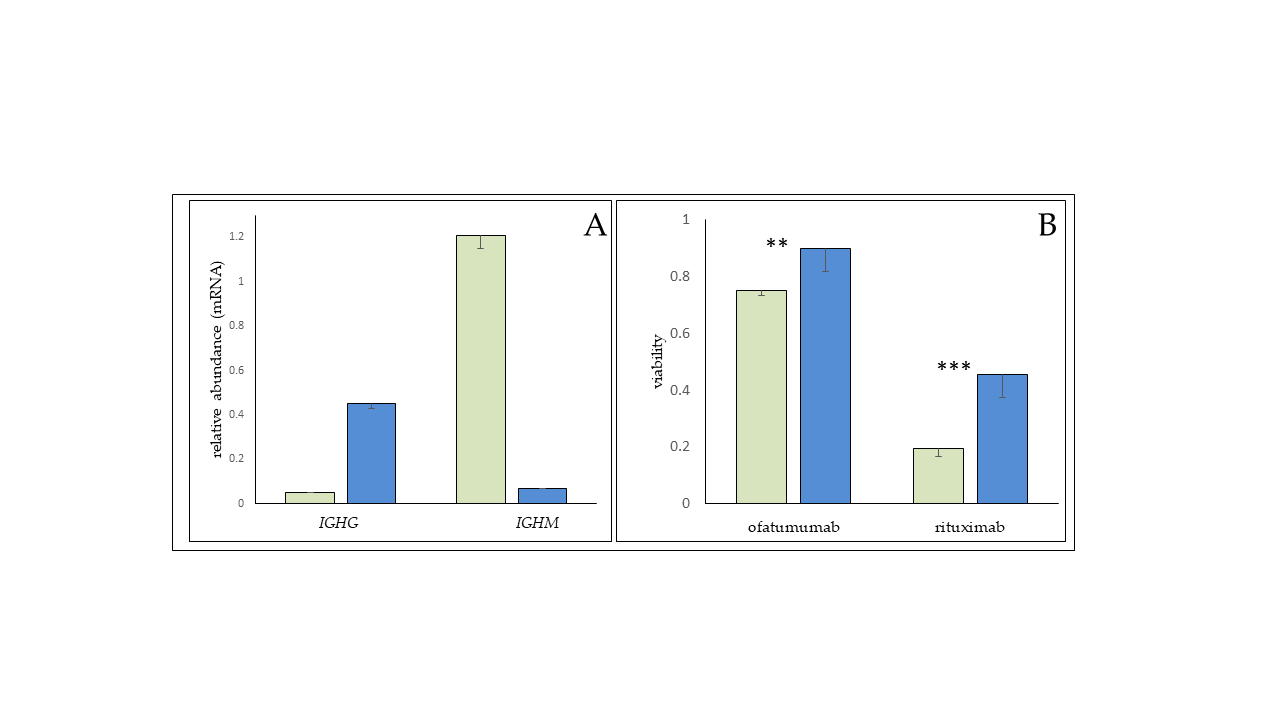

Supplement: Supplementary file 1 [file cells-12-01574-s001.zip › Figure S7-Separation by Ig isotype.tif]

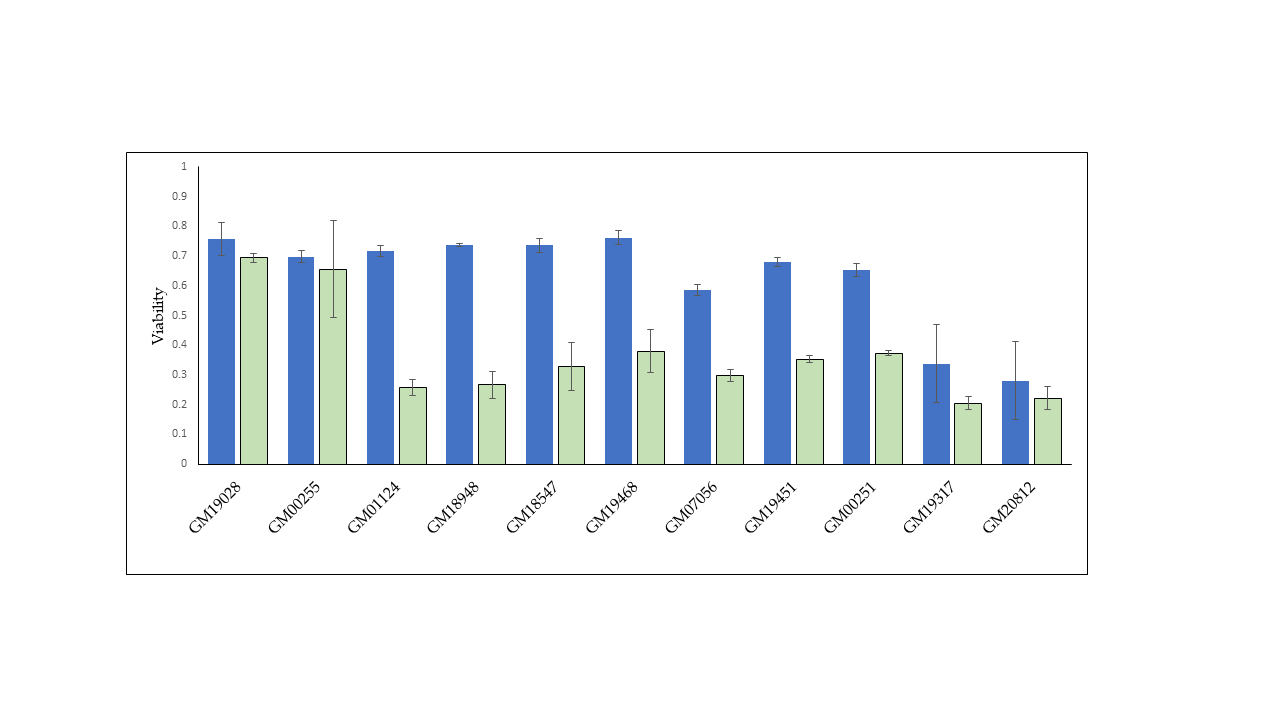

Supplement: Supplementary file 1 [file cells-12-01574-s001.zip › Figure S8-prednisolone sensitivity.tif]

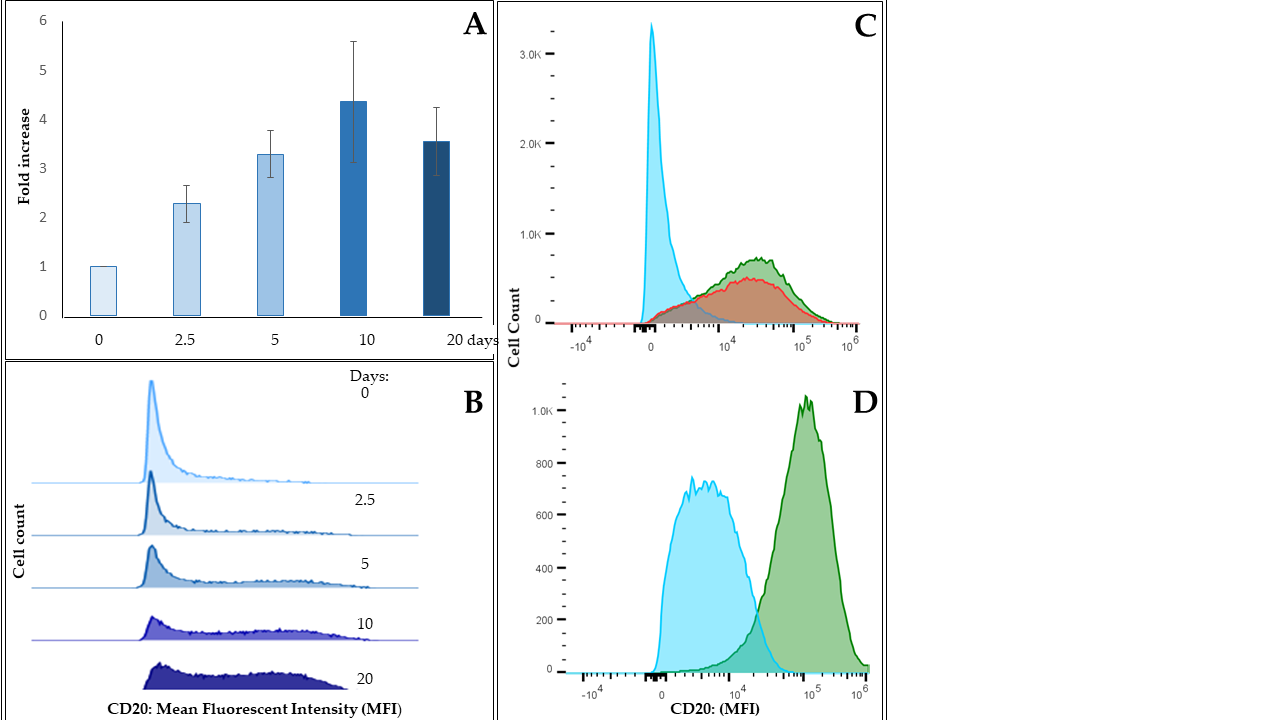

Supplement: Supplementary file 1 [file cells-12-01574-s001.zip › Figure S9-pred CD20 expression.tif]
